# Diazirine-functionalized mannosides for photoaffinity labeling: trouble with FimH

**DOI:** 10.3762/bjoc.14.163

**Published:** 2018-07-24

**Authors:** Femke Beiroth, Tomas Koudelka, Thorsten Overath, Stefan D Knight, Andreas Tholey, Thisbe K Lindhorst

**Affiliations:** 1 Otto Diels Institute of Organic Chemistry, Christiana Albertina University of Kiel, Otto-Hahn-Platz 3/4, 24118 Kiel, Germany; 2 Systematic Proteomics & Bioanalytics, Institute for Experimental Medicine, Christiana Albertina University of Kiel, Niemannsweg 11, D-24105 Kiel, Germany; 3 Department of Cell and Molecular Biology, Uppsala University, Uppsala Biomedical Centre, P.O. Box 596, S-751 24 Uppsala, Sweden

**Keywords:** diazirines, docking, FimH, lectin ligands, mannosides, mass spectrometry, photoaffinity labelling

## Abstract

Photoaffinity labeling is frequently employed for the investigation of ligand–receptor interactions in solution. We have employed an interdisciplinary methodology to achieve facile photolabeling of the lectin FimH, which is a bacterial protein, crucial for adhesion, colonization and infection. Following our earlier work, we have here designed and synthesized diazirine-functionalized mannosides as high-affinity FimH ligands and performed an extensive study on photo-crosslinking of the best ligand (mannoside **3**) with a series of model peptides and FimH. Notably, we have employed high-performance mass spectrometry to be able to detect radiation results with the highest possible accuracy. We are concluding from this study that photolabeling of FimH with sugar diazirines has only very limited success and cannot be regarded a facile approach for covalent modification of FimH.

## Introduction

**Figure 1:**
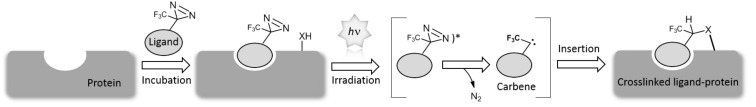
Principle of photoaffinity labeling of proteins with diazirine derivatives. Photolabile ligands are complexed with the receptor protein and the following photochemical excitation of the complex leads to formation of a reactive carbene after extrusion of nitrogen and a crosslinked product after insertion reaction; X = e.g., NH, O, CH_2_.

The investigation of the interactions between proteins and their ligands, such as lectins and carbohydrates, is of fundamental importance for the understanding of many biological processes. Several different methodologies are used for the elucidation of ligand–protein interactions. In addition to X-ray crystallography, studies in solution add valuable information in molecular recognition studies as they take molecular dynamics as well as solvent effects into consideration. In the latter respect, photoaffinity labeling has evolved as a useful tool for studies under physiological conditions [1–4]. Photoaffinity labeling requires a ligand equipped with a photolabile group, which can be converted into a highly reactive intermediate upon irradiation with light of an appropriate wavelength. This technique involves incubation of the photolabile ligand with the target protein (receptor) and irradiation of the ligand–protein complex to form an excited intermediate, which eventually leads to a covalently crosslinked ligand–receptor conjugate, which has to be identified by mass spectrometry (Figure 1).

Three widely used photoreactive groups are aryl azides, benzophenones and diazirines. They differ with respect to their steric properties, photochemistry, and reactivity. In order to be applied in a biological environment, the wavelength of the light required for activation of the photolabile ligand has to be biocompatible. Furthermore, small photophores are desirable. Diazirines meet these requirements particularly well. The diazirine photophore is small and its photoactivation is possible at wavelengths around 350 nm and thus does not perturb protein structures. According to the literature, irradiation of a diazirine-functionalized ligand leads to a reactive carbene which can insert into OH, NH, or CH groups of a protein in a fast reaction [5–6]. Further advantages of the diazirine group are its robustness at different pH values and its stability against nucleophiles. However, besides desired insertion reactions, carbenes can undergo unwanted side reactions, in particular olefin formation through abstraction of α-H atoms. Therefore, aryl(trifluoromethyl)diazirines were introduced, which lack hydrogen atoms in α-position of the diazirine function and consequently do not form olefins. Thus, aryl(trifluoromethyl)diazirines have become reagents of first choice for photolabeling studies [7–8].

Over several years it has been our goal, to exploit diazirine-labeled mannopyranosides for protein labeling. In the course of this work, we have gradually improved our target design and synthetic procedures [9–11] with the aim to eventually address the bacterial lectin FimH. Finally, back in 2010, we analyzed photolabeling of the octapeptide angiotensin II with three different sugar diazirines using mass spectrometry and then also detected photolabeling of FimH with the same three mannosides. However, we could not measure the labeling product with full accuracy [11]. Although these results were even recognized in a recent review [12], they unfortunately did not help to consolidate our projects on photoaffinity labeling of FimH, but instead this methodology remained problematic in our hands. Until to date, no reliable ligands for photolabeling of FimH are available.

We reasoned that high performance mass spectrometry-based proteomics could lead to more robust success in our approach to employ tailor-made sugar ligands for FimH labeling. Here, we report on the difficulties of FimH labeling even when computer-aided design and synthesis of photolabile FimH were combined with optimized photolabeling conditions and high-end mass spectrometry.

## Results and Discussion

**Figure 2:**
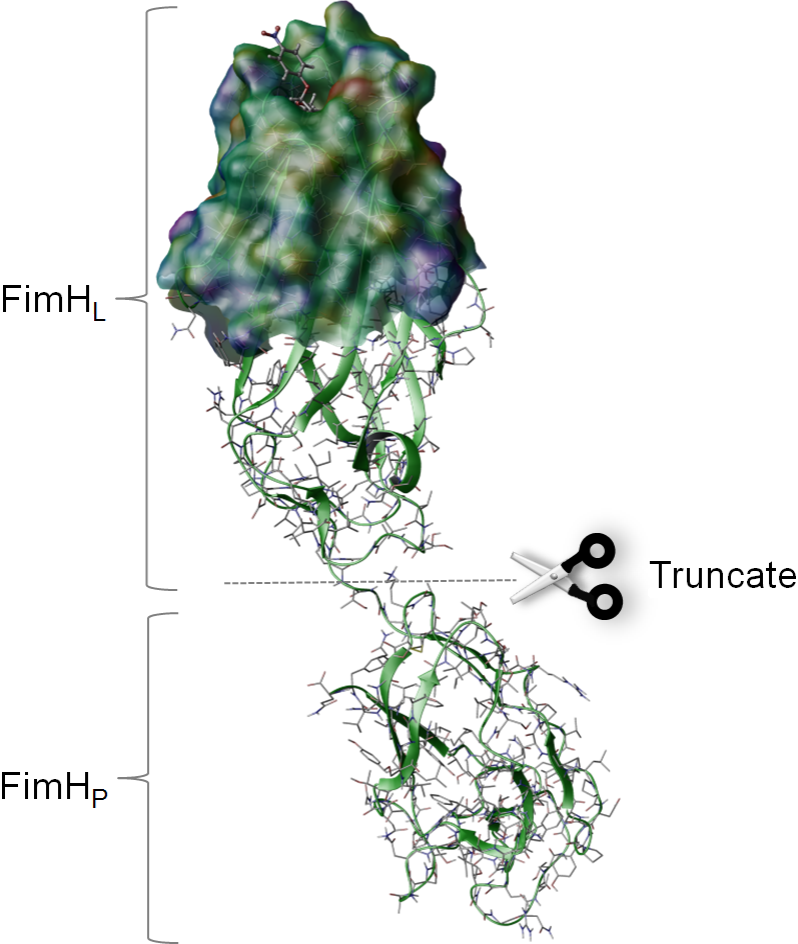
FimH crystal structure (pdb code 1KLF) with docked *p*-nitrophenyl α-D-mannopyranoside (**1**, *p*NPMan). FimH is a two-domain protein comprising a lectin domain (FimH_L_) with the carbohydrate binding site (top) and a pilin domain (FimH_P_, bottom) that anchors the lectin onto the fimbrial shaft. For photoaffinity labeling, a truncated version of the protein (FimH_tr_) was used. The carbohydrate binding site is depicted as Connolly surface and colored according to the electrostatic potential. The picture was generated with glide and rendered with maestro, both implemented in Schrödinger software.

FimH is a fimbrial lectin found at the tips of adhesive organelles (type 1 fimbriae), which are projecting from the surface of enterobacteriaceae such as *E. coli*. Fimbriae mediate firm attachment (adhesion) of bacteria to the glycosylated surface of their target cells and constitute important virulence factors in bacterial infection such as urinary tract infection [13–16]. Hence, FimH is an interesting target protein in medicinal chemistry and proteomics [17–18]. It is a two-domain protein comprising a lectin domain FimH_L_ hosting the α-D-mannose-specific carbohydrate binding site and a pilin domain FimH_P_ connecting the protein to the fimbrial shaft (Figure 2).

**Figure 3:**
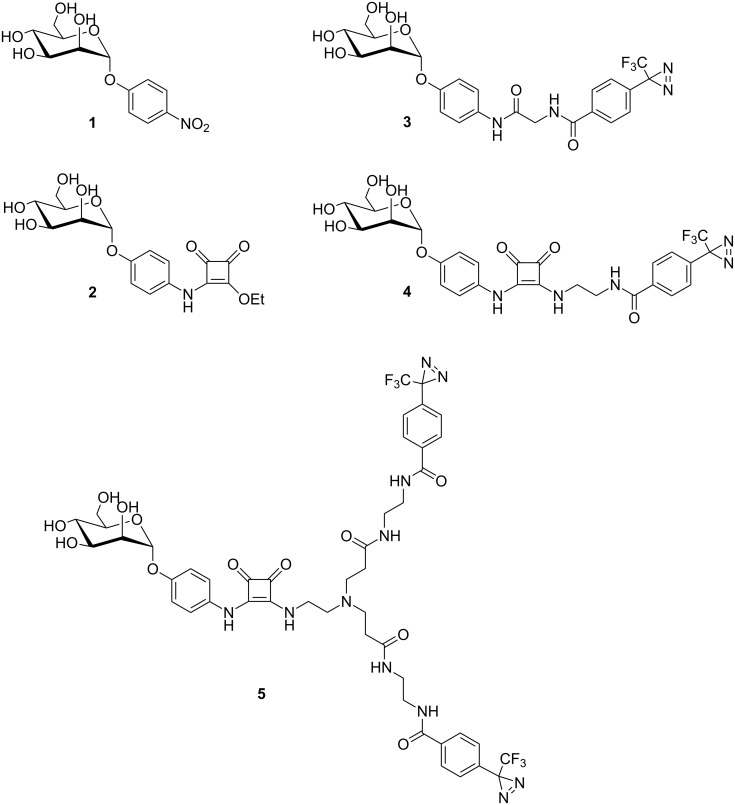
Based on the structure of the known FimH ligands **1** and **2**, three photolabile α-D-mannosides, **3**–**5**, were considered for FimH labeling. In **3** and **4** the distance between the diazirine functional group and the anomeric center of the mannoside are varied. In **5** a bis-diazirine functionalization is suggested to increase labeling probability.

Complexation of α-D-mannopyranoside ligands involves the entire mannoside glycon moiety whereas the aglycon portion sticks out of the binding site, undergoing interactions with the protein surface, which add to affinity. Especially CH–π or π–π interactions of a sugar ligand with the side chains of Y48 and Y137, called the “tyrosine gate” [19–20], are known to considerably increase the affinity of a specific mannoside for FimH. Consequently, α-D-mannopyranosides having an aromatic aglycon portion such as *p*-nitrophenyl α-D-mannopyranoside (**1**) and the squaric acid derivative **2** [19] (Figure 3) were identified as FimH ligands with relative high affinity (low μmolar range). Based on this knowledge, we proposed the three diazirine-labeled mannosides **3**–**5** as photolabile ligands of FimH and evaluated their potential affinity by computer-aided docking.

### Docking studies

Mannosides **3**–**5** (Figure 3) were docked into the FimH carbohydrate binding site using FlexX as implemented in Sybyl 6.9 [21–23]. Ligand structures were minimized using the Tripos force field and 30 different conformers of each ligand were docked into two different X-ray structures of FimH (for details see Supporting Information File 1). These two crystal structures differ in the conformation of the tyrosine gate, formed by Y48 and Y137 positioned at the entrance of the carbohydrate binding site. They are flexible and can be more distant to one another (“open gate”) or closer together (“closed gate”) [20]. Both conformations were considered for the docking studies. For each docked conformation, a scoring value is obtained that correlates with the affinity of the ligand to the carbohydrate binding site. A more negative value predicts better binding. Scores obtained for mannosides **3** and **4** suggest a high affinity for FimH, surpassing that of *p*NPMan **1** for both protein conformations tested (Table 1). (Diazirines cannot be tested in bacterial adhesion–inhibition assays due to their light sensitivity.)

**Table 1:** FlexX scoring values for photolabile mannosides **3**–**5** in comparison with methyl α-D-mannopyranoside (MeMan) and *p*NPMan **1** for the open and closed gate crystal structure of FimH (PDB 1KLF and 1UWF, respectively).

Ligand	FlexX scoring value open gate	FlexX scoring value closed gate

MeMan	−22.5	−23.3
**1**	−24.9	−27.4
**3**	−34.7	−36.2
**4**	−28.6	−34.0
**5**	–	−18.8

**Figure 4:**
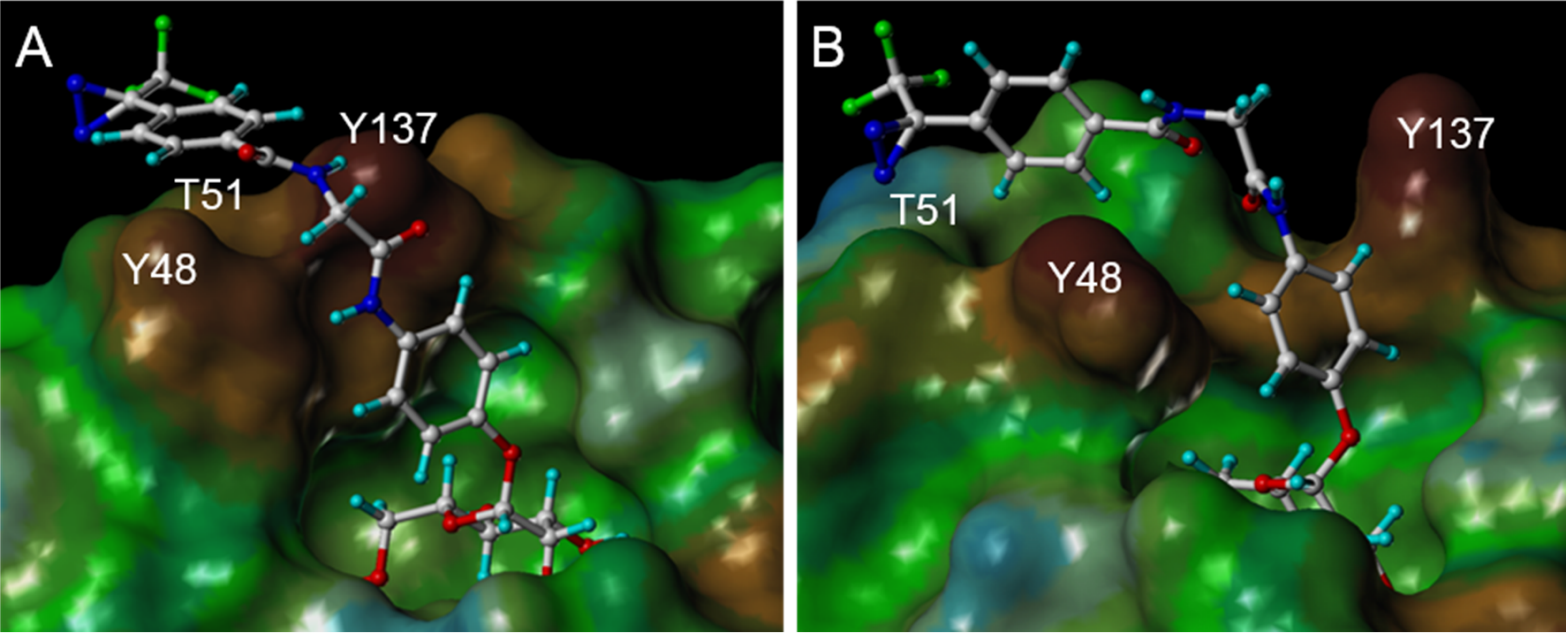
Connolly representation of photolabile α-D-mannoside **3** in the closed gate (A, PDB code 1UWF) and open gate crystal structure of FimH (B, PDB code 1KLF) [24]. The surface is colored according to the lipophilic potential, where brown reflects more hydrophobic and blue more hydrophilic amino acid residues [25]. Amino acid residues Y48 and Y137, forming the “tyrosine gate” are highlighted as well as T51, which is a good candidate for photolabeling.

The bivalent ligand **5**, on the other hand, seems to be sterically too demanding to allow good complexation with the carbohydrate binding site of FimH; mainly unspecific interactions with the surface of FimH were predicted in this case. Thus, synthesis of **5** was not undertaken. In contrast, the diazirine **3**, with scoring values of −34.7 (open gate) and −36.2 (closed gate), is predicted as suitable FimH ligand with high affinity. Additionally, docking suggests that the diazirine function of **3** is positioned in close proximity to the protein surface, making a specific insertion reaction of the carbene formed after irradiation very likely (involving for example Y48 or T51, Figure 4). Docking of mannoside **4** also delivered good scoring values and therefore both photolabile ligands **3** and **4** were synthesized.

### Synthesis of photolabile mannosides

**Scheme 1:**
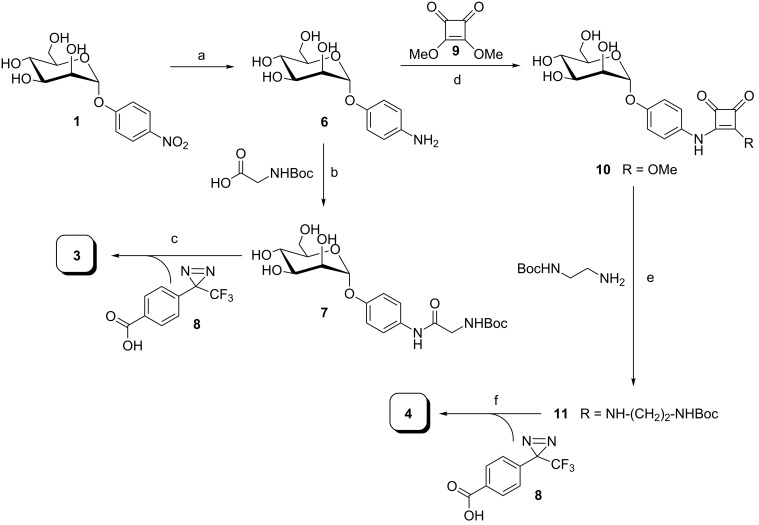
Synthesis of photolabile α-D-mannosides **3** and **4**. a) H_2_, Pd-C, methanol, rt, 6 h, 94%; b) HATU, DIPEA, dry DMF, N_2_, rt, overnight, quant.; c) 1. 50% TFA in water, rt, 3 h; 2. HATU, **8**, DIPEA, dry DMF, N_2_, rt, 14 h, 19% (over 2 steps); d) dry methanol, rt, 16 h, 59%; e) Et_3_N, dry methanol, rt, 15 h, 92%; f) 1. 50% TFA in water, rt, 3 h; 2. HATU, **8**, DIPEA, dry DMF, N_2_, rt, overnight, crude product (68% over 2 steps).

The synthesis of the photolabile mannosides **3** and **4** started from *p*-nitrophenyl α-D-mannopyranoside (**1**), which was first reduced to the corresponding amine **6** [26–27] by catalytic hydrogenation (Scheme 1). HATU-mediated peptide coupling with Boc-protected glycine under basic conditions led to **7**. After removal of the Boc protecting group using trifluoroacetic acid in water, the resulting crude product was subjected to a subsequent peptide-coupling reaction employing the carboxy-functionalized diazirine **8**.

Diazirine **8** was prepared in a nine-step synthesis according to the literature [28]. For the synthesis of ligand **4**, mannoside **6** was first converted into the squaric acid monoester **10** employing squaric acid diester **9**. The monoester **10** was reacted with *N*-Boc-ethylendiamine to obtain the squaric acid diamide **11**. Then removal of the Boc protecting group with trifluoroacetic acid followed by peptide coupling with the diazirine **8** led to target molecule **4**. However, purification of the photolabile mannosides is problematic due to their poor solubility and the light sensitivity of the diazirine moiety. Therefore, the products often contain minor impurities after chromatography resulting from light-induced activation of the diazirine and insertion reaction. This can be monitored by ^19^F NMR spectroscopy showing the typical diazirine CF_3_ signal around −68 ppm [29]. Whereas mannoside **3** was received in good purity, compound **4** was always obtained with contaminations. Thus, **3** was the preferred ligand for the following photolabeling experiments. Results obtained with mannoside **4** were less promising and are not reported here. After all, also modeling had suggested that **3** has a higher affinity for FimH than **4**.

To test carbene formation, mannoside **3** was irradiated at 345 nm in 1:1 acetonitrile/water as well as in 1:1 DMSO/water mixtures with 4-hydroxybenzyl alcohol as the simplest tyrosine mimic. Mass-spectrometric analysis indicated the desired crosslinked product as well as insertion into water in both cases (cf. Supporting Information File 1, Table S5). Hence, carbene formation upon irradiation of diazirine **3** was ensured and thus we continued further labeling studies with more complex substrates.

### Irradiation of mannoside **3** and model peptides

In analogy to our earlier work, we first used a series of model peptides (M2, M3, M7, M8, T3, and S17, cf. Table 2) for the photolabeling experiments with mannoside **3**. These model peptides were chosen rather arbitrarily as we have frequently employed them to optimize mass spectrometric procedures. Also, we were interested to test, if threonine or tyrosine residues, respectively, would lead to better labeling efficiency. Because mannoside **3** is poorly water soluble, it was dissolved in methanol and then added to the peptides in a 1:1 ratio. After irradiation with UV light, two new signals were detected in nano-LC–ESIMS experiments in comparison to the original peptide spectra (Table 2). It should be noted that whereas we could monitor photodecomposition of the photolabels by ^19^F NMR spectroscopy (cf. [29]), we could not observe labeled products by fluorine NMR. This is presumably due to the low labeling efficiency as in the literature ^19^F NMR spectra were obtained with proteins having ^19^F-labeled amino acids incorporated [30].

**Table 2:** ESIMS data of peptide labeling with ligand **3** (cf. Supporting Information File 1 for all details).

Peptide	Peptide mass (M)	Detected masses after labeling (*m*/*z*)

ILMEHIHKL (M2)	1132.6499	531.1584 [M + H]^+ a^, 832.4043 [M + 2H]^2+ b^ (Figure 5)
YLLPAIVHI (M3)	1037.6274	531.1584 [M + H]^+ a^, 784.8971 [M + 2H]^2+ b^ (Figure S11, Supporting Information File 1)
EIAMATVTALR (M7)	1174.6380	531.1586 [M + H]^+ a^, 853.4019 [M + 2H]^2+ b^ (Figure S14, Supporting Information File 1)
ETIGEILKK (M8)	1029.6071	531.1587 [M + H]^+ a^, 780.8868 [M + 2H]^2+ b^ (Figure S17, Supporting Information File 1)
EGHIARNCRA (T3)	1125.5462	531.1588 [M + H]^+ a^, no adduct observed (Figure S20, Supporting Information File 1)
RPQYAEASWNAR (S17)	1447.6957	531.1585 [M + H]^+ a^, 989.9307 [M + 2H]^2+ b^ (Figure S21, Supporting Information File 1)

^a^Insertion product of **3** − N_2_ + H_2_O; ^b^Adduct of **3** − N_2_ + H_2_O to the peptide.

**Figure 5:**
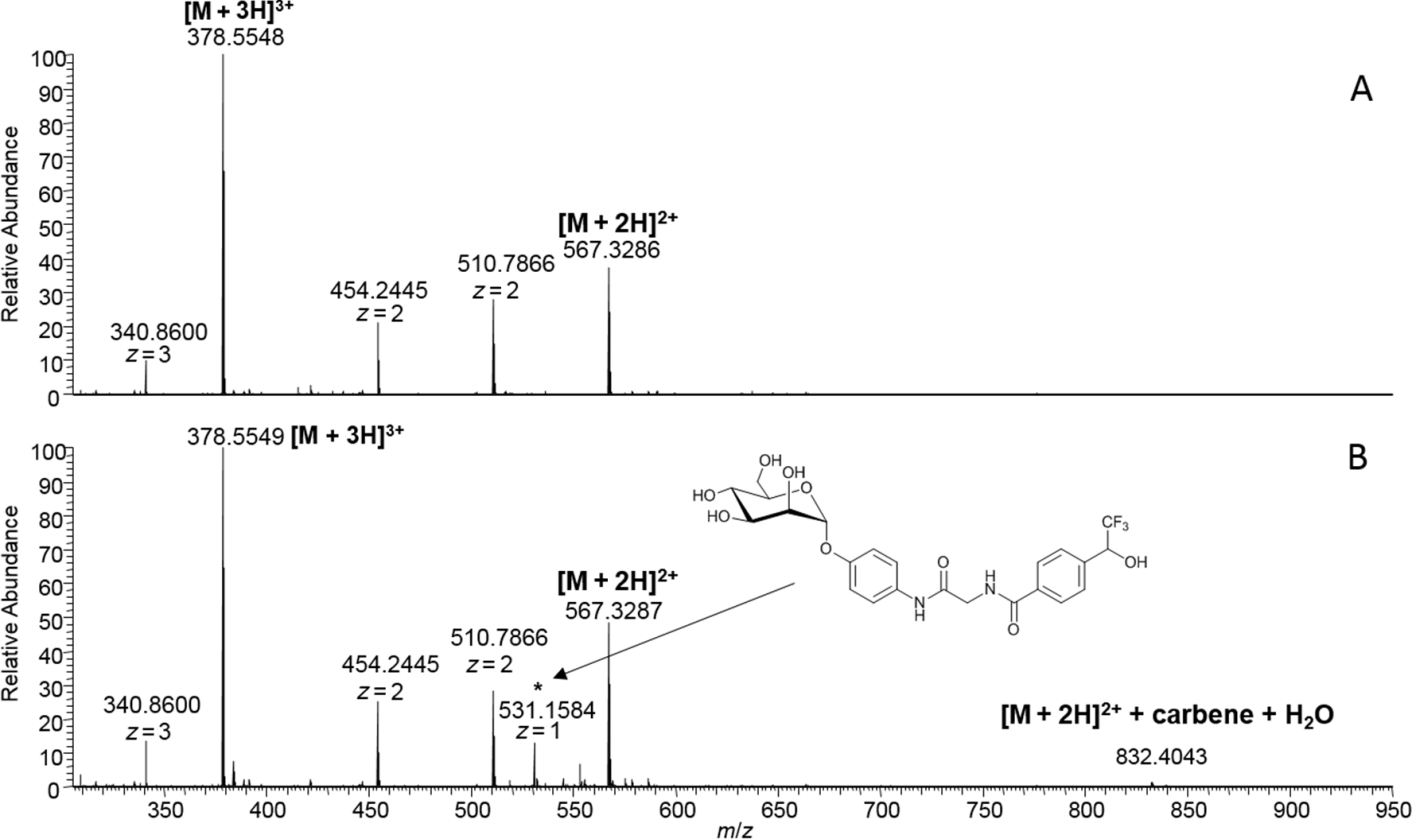
ESIMS spectra of peptide M2 before (A) and after photoreaction (B) with mannoside **3**.

Disappointingly, instead of crosslinking, we observed two different other products in all cases. As an example, for peptide M2 one peak at *m/z* 531.1584 ([M + H]^+^) could be assigned to the ligand **3** that had reacted with a water molecule instead of the peptide (Figure 5). The second signal of low intensity in this spectrum at *m*/*z* 832.4043 corresponds to the peptide mass plus hydrolyzed carbene. However, MS/MS fragmentation of both the unmodified peptide (567.3281)^2+^ and the allegedly modified peptide (832.4043)^2+^ exhibited the same b- and y-ion series (cf. Supporting Information File 1, Figure S10). Accordingly, the low intensity peak is most likely the result of non-covalent binding of hydrolyzed **3** to the peptide M2 rather than a covalent modification at one of the peptide’s side chains. The same situation was found for all other peptides irradiated with the photolabile ligand **3** (cf. Supporting Information File 1).

### Irradiation of mannoside **3** and FimH-truncate

**Figure 6:**
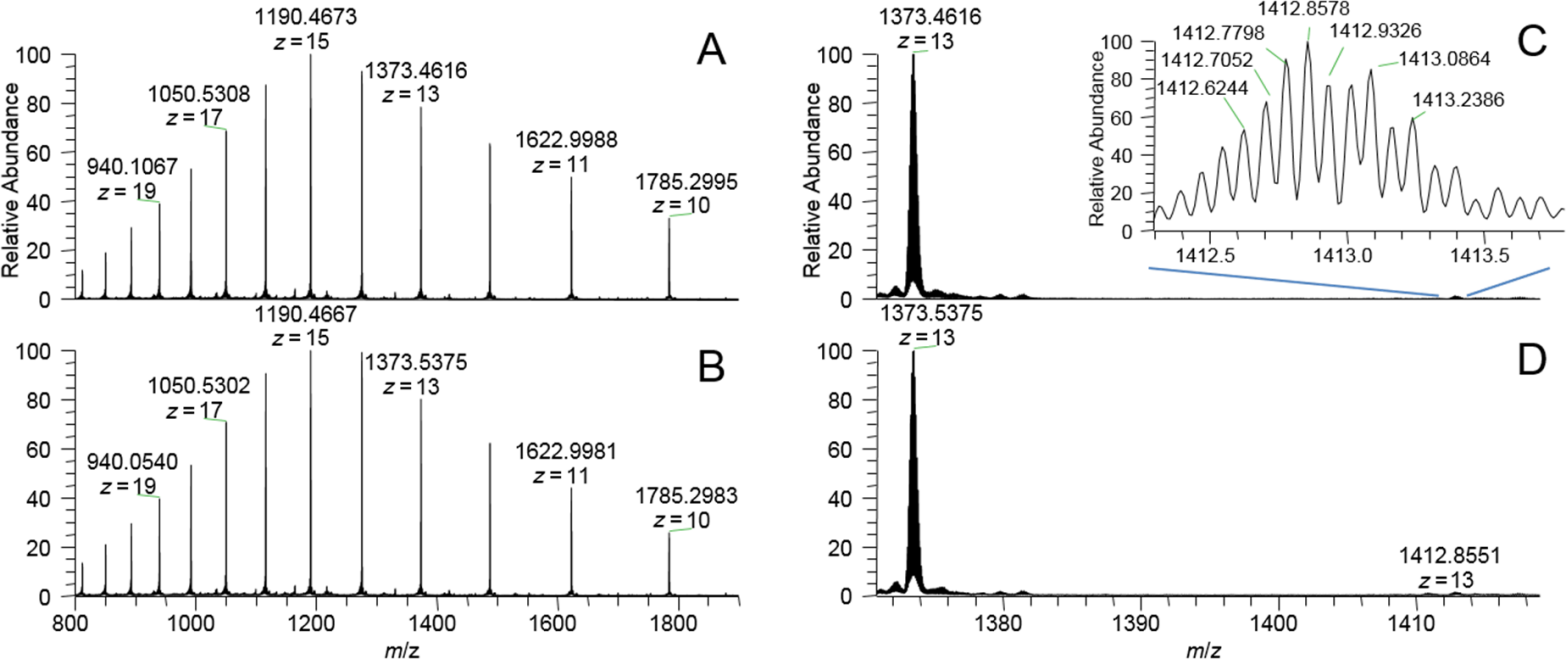
Intact protein ESIMS spectra of FimH_tr_ labeling with diazirine derivative **3** with (A) 50% acetonitrile or (B) 10% DMSO additive. (C) Minor peak at 1412.8578, as part of spectrum A, representing the covalently bound label. Zoom into the signal at charge state +13. Labeling yield calculated from peak intensities compared to the corresponding signal of unlabeled peptide: 1.41% (50% acetonitrile). (D) Minor peak at 1412.8551, as part of spectrum B, representing the covalently bound label. Labeling yield calculated from peak intensities compared to corresponding signal of unlabeled peptide: 1.55% (10% DMSO). The theoretically expected mass of labeled FimH_tr_ is 1412.9333 Da. The fact that the most abundant isotope was not 1412.9333, but 1412.8578 and 1412.8551 Da is simply due to the high signal to noise ratio of the spectra as a result of low labeling efficiency.

Whereas we first expected that it would be easier to photolabel peptides than FimH, after the failure with photolabeling of the model peptides we were hoping that labeling of FimH would be more successful, as our ligand was designed to bind to FimH. We reasoned that complexation of the photolabile mannoside **3** and FimH could support the desired crosslinking reaction. Thus, the high affinity of **3** for FimH would facilitate insertion of the carbene, which is formed after irradiation, into OH, NH or CH groups, respectively, in proximity of the lectin’s carbohydrate binding site. We were hoping that after our initial success in 2010 [11], high performance MS analysis would lead to results of higher accuracy. Indeed, we could detect FimH_tr_ labeling after irradiation with **3**, however, photolabeling was not at all efficient. Only after extensive and tedious optimization of reaction conditions, a new peak was observed by mass spectrometry on the intact protein level (Figure 6). For this, the photolabile mannoside **3** was incubated with FimH truncate (FimH_tr_) in buffer and 50% ACN or 10% DMSO, respectively, and irradiated with UV light.

The theoretically most abundant isotope peak of unbound FimH_tr_ (1373.4608 Da, *z* = 13) fits to the most abundant experimentally observed mass for unbound FimH_tr_ with 1373.4616 Da (Figure 6A) and 1373.5375 Da (Figure 6B) with small deviations. The presence of a new peak at 1412.8578 Da (*z* = 13, Figure 6C) and 1412.8551 Da (*z* = 13, Figure 6D), respectively, corresponded very well with insertion of the photolabile mannoside **3**. In this case, non-covalent bonding of the photolabile mannoside **3** to the substrate (FimH_tr_) could be ruled out, as this would have led to a significantly higher mass according to the intact label **3** with a relative mass shift of ≈1.38 (at *z* = 13). In addition, under the LC–ESIMS conditions applied here, e.g., low pH and use of organic eluents in ion-paring reversed-phase chromatography, as well as under the ESI conditions employed, non-covalent bonding of the photolabile mannoside to FimH is unlikely to be observed. Due to the low labeling yield obtained, all our attempts to identify the specific position of FimH labeling by peptic digestion of the labeled protein and following MS/MS analysis were unsuccessful.

## Conclusion

In continuation of our earlier work, we have employed an advanced interdisciplinary approach to identify diazirine-labeled mannoside ligands for facile and reliable photolabeling of the bacterial lectin FimH. We could indeed show photolabeling of the protein with the designer mannoside **3** with much higher accuracy than before. However, a great excess of ligand was needed and only traces of labeled FimH were observed. Due to the high-performance mass spectrometry which was employed here, this result can be considered specific. However, it was not possible to identify the site of labeling due to the low labeling yield. Photolabeling of FimH with sugar diazirines remains difficult and thus this approach is not very likely to be developed into a standard method for FimH labeling. As we wish to eventually control bacterial adhesion by crosslinking of functional molecules to the adhesin (FimH), we will in the future utilize alternative labeling chemistry to efficiently target FimH.

## Experimental

### Peptides and proteins

For photolabeling experiments model peptides from Biosynthan (Berlin) were used: ILMEHIHKL (M2), YLLPAIVHI (M3), EIAMATVTALR (M7), ETIGEILKK (M8), EGHIARNCRA (T3), and RPQYAEASWNAR (S17). Furthermore, FimH truncate (FimH_tr_) was used, which was expressed and purified in the laboratory of the S. D. Knight according to [31]. FimH_tr_ has the following amino acid sequence: FACKTANGTAIPIGGGSANVYVNLAPVVNVGQNL-VVDLSTQIFCHNDYPETITDYVTLQRGSAYGGVLSNFSG-TVKYSGSSYPFPTTSETPRVVYNSRTDKPWPVALYLT-PVSSAGGVAIKAGSLIAVLILRQTNNYNSDDFQF-VWNIYANNDVVVPTGGHHHHHH.

### Docking studies

Computer-aided docking studies were performed to evaluate the binding affinity of FimH ligands using FlexX flexible docking and consensus scoring, both implemented in Sybyl 6.9, as described earlier [19]. Two published X-ray structures of FimH (PDB codes 1KLF and 1UWF) were considered. During docking, the receptor was held fixed whereas the ligand was allowed to change its conformation. All calculations were done with the Tripos force field.

### Peptides

To 500 pmol of a model peptide (cf. Table 1) dissolved in 5 µL double distilled water (18 MΩ) with 5% acetonitrile (ACN), 500 pmol of ligand solution (**3** in 12.9 µL methanol) was added and incubated for 10 min at 37 °C and 100 rpm. Subsequently, the solution was irradiated under ice cooling with a medium pressure mercury vapor lamp for 10 min. Afterwards, 50 µL double distilled water were added and 2 µL of this solution given to 20 µL of a 0.1% trifluoroacetic acid (TFA) solution, desalted with a C18 µZipTip and spotted on a MALDI-plate together with 5 mg/mL α-cyano-4-hydroxycinnamic acid (HCCA) matrix (70% ACN in 0.1% TFA solution in double distilled water). MALDI–TOF MS was performed on a AB 5800 MS (AB Sciex, Darmstadt, Germany) in positive ion mode.

### FimH_tr_

FimH_tr_ (3.36 nmol) was dissolved in PBS buffer (pH 7.8). Ligand **3** was dissolved in ACN or DMSO. The ratio ligand to protein was 70:1. The total volume of the reaction was 11 µL either with 50% ACN or 10% DMSO. Ligand and protein were combined and incubated for 10 min at 37 °C and 100 rpm. Afterwards the solution was irradiated under ice cooling with a medium pressure mercury vapor lamp for 10 min. The protein solution was then investigated by ESI mass spectrometry.

### LC–MS methods

Nano-LC–MS was performed on the U3000 nano-LC-UV system (Dionex, Idstein, Germany) coupled online to a LTQ Orbitrap Velos mass spectrometer equipped with LTQ Tune Plus 2.7.0 and XCalibur 2.2 (all Thermo Fisher Scientific). The column oven was set to 30 °C and the UV trace was monitored at a wavelength of 214 nm. The modified FimH_tr_ (10 pmol) was desalted on a C4 trap column (Acclaim PepMap C-4, 300 µm i.d. × 5 mm, 5 µm, 300 Å, Thermo Fisher Scientific) using a flow rate of 30 µL/min. After 5 min, the analytes were eluted with a flow rate of 300 nL/min onto an analytical monolithic column (ProSwift RP-4H, 100 µm × 250 mm, Thermo Fisher Scientific) using eluent A (0.05% formic acid (FA)) and eluent B (80% ACN in 0.04% FA). A gradient from 5 to 95% B was applied over 30 min followed by a 10 min washing step at 95% B and a 15 min equilibration step at 5% B. MS data were recorded in MS full scan mode between 400 and 2000 *m*/*z* using 445.120025 as a lock mass. Data were recorded at a resolution of 60,000 in profile mode. To obtain better quality MS data, no MS/MS data were acquired. Raw files were opened in XCalibur 2.2 and spectra during which FimH_tr_ was observed were averaged. Theoretical spectra of FimH_tr_ were compared to the experimental spectra using IDCalc (version 0.3). Experimental spectra were calculated in profile mode using a charge state of 13 and at a resolution of 35,000 (the actual resolution observed at *m*/*z* of 13 for FimH_tr_) and an elemental composition of truncated and his-tagged FimH with a disulfide bond (C_803_H_1213_O_242_N_217_S_2_). The relative peak abundances were extracted and compared to the theoretical spectra. A change in molecular composition upon addition of the diazirine **3** (C_23_H_23_F_3_N_2_O_8_) was appended to the elemental composition of FimH_tr_.

### Methods and materials for synthesis

TLC was performed on silica gel GF_254_ (Merck). Spots were visualized under UV light and by charring with 10% sulfuric acid in ethanol and subsequent heating. Flash column chromatography was performed on silica gel 60 (230–400 mesh, particle size 40–63 µm, Merck). All used solvents were distilled. NMR spectra were recorded on Bruker DRX 500 or Bruker Avance 600 instruments at 300 K. Chemical shifts are relative to the solvent peak of MeOD (3.35 ppm and 4.78 ppm for ^1^H, 49.3 for ^13^C). Assignments of the signals were done using 2D experiments (COSY, HSQC and HMBC). IR spectra were measured with a Perkin Elmer FT IR Paragon 1000 (KBr). Optical rotation values were determined with a Perkin-Elmer polarimeter (589 nm, length of cuvette: 1 dm). MS analysis of synthetic products were carried out with a MALDI–TOF mass spectrometer Bruker Biflex III with 19 kV acceleration voltage (matrix: 2,5-dihydroxybenzoic acid) and with a ESI–TOF MS spectrometer Applied Biosystems Mariner ESI–TOF 5280. UV data were obtained with the Perkin-Elmer UV–vis spectrometer Lambda 14 at 25 °C. All reactions and purification steps of diazirine compounds were performed in the dark.

***N*-{2-Oxo-2-[(4-(α-D-mannopyranosyloxy)phenyl)amino]ethyl}-4-(3-trifluoromethyl-3*H*-diazirin-3-yl)benzamide (3).** The glycoamino acid **7** (52.8 mg, 160 µmol) was dissolved in distilled water which contained 50% TFA (5 mL). The solution was then stirred at room temperature for 3 h. After complete conversion, the solvent was removed under reduced pressure, co-distilled with toluene and neutralized with basic ion exchange resin in methanol. The crude product was then dried together with diazirine **8** [28] (27.6 mg, 120 µmol) and HATU (83.7 mg, 220 µmol) for 1 h under vacuum. Subsequently this was mixture dissolved in dry DMF (5 mL) and DIPEA (20.0 µL, 140 µmol). The solution was stirred at room temperature for 14 h. The solvent was removed under reduced pressure and the residue purified by flash column chromatography (ethyl acetate/methanol 5:1). The product was isolated as a colorless solid (17.2 mg, 30.0 µmol, 19%); *R*_f_ = 0.76 (ethyl acetate/methanol 2:1); UV–vis (MeOH) λ_max_: 345 nm; FTIR (KBr) 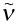: 3317, 2927, 2503, 2093, 1671, 1637, 1550, 1511, 1224, 1015, 940, 681 cm^−1^; ^1^H NMR (500 MHz, MeOH-*d*_4_) δ 7.99 (d, ^3^*J* = 8.5 Hz, 2H, CH_aryl_-2‘,6‘), 7.48 (d, ^3^*J* = 9.1 Hz, 2H, CH_aryl_-2,6), 7.36 (d, ^3^*J* = 8.5 Hz, 2H, CH_aryl_-3‘,5‘), 7.08 (d, ^3^*J* = 9.1 Hz, 2H, CH_aryl_-3,5), 5.43 (d, ^3^*J*_1,2_ = 1.7 Hz, 1H, H-1), 4.18 (s, 2H, CH_2-Glycin_), 3.99 (dd, ^3^*J*_1,2_ = 1.7 Hz, ^3^*J*_2,3_ = 3.4 Hz, 1H, H-2), 3.89 (dd, ^3^*J*_3,2_ = 3.4 Hz, ^3^*J*_3,4_ = 9.4 Hz, 1H, H-3), 3.76 (dd, ^3^*J*_6,5_ = 2.5 Hz, ^2^*J*_6,6‘_ = 11.9 Hz, 1H, H-6), 3.73 (m_c_, 1H, H-4), 3.71 (dd, ^3^*J*_6‘,5_ = 5.1 Hz, ^2^*J*_6‘,6_ = 11.9 Hz, 1H, H-6‘), 3.61 (ddd, ^3^*J*_5,6_ = 2.5 Hz, ^3^*J*_5,6‘_ = 5.1 Hz, ^3^*J*_5,4_ = 9.8 Hz, 1H, H-5) ppm; ^13^C NMR (126 MHz, MeOH-*d*_4_) δ 171.6 (1C, CN_2_), 169.5 (1C, C=O), 169.2 (1C, C=O), 154.7 (1C, C_aryl_-4), 136.7 (1C, C_aryl_-1‘), 134.1 (1C, C_aryl_-1), 133.3 (1C, C_aryl_-4‘), 129.3 (2C, CH_aryl_-2‘,6‘), 127.7 (2C, CH_aryl_-3‘,5‘), 122.9 (2C, CH_aryl_-2,6), 121.3 (1C, q, *J* = 274.9 Hz, CF_3_), 118.2 (2C, CH_aryl_-3,5), 100.6 (1C, C-1), 75.4 (1C, C-5), 72.4 (1C, C-3), 72.0 (1C, C-2), 68.4 (1C, C-4), 62.7 (1C, C-6), 44.4 (1C, CH_2-glycine_) ppm; ^19^F NMR (471 MHz, MeOH-*d*_4_) δ −66.87 (s) ppm; ESIMS *m*/*z*: 579.12 [M + K]^+^, 563.14 [M + Na]^+^, 535.13 [(M + Na) − N_2_]^+^; HRESIMS *m*/*z*: [M + H]^+^ calcd. for C_23_H_23_F_3_N_4_O_8_, 541.15408; found, 541.15424.

**{*N*-[4-(α-D-Mannopyranosyloxy)phenyl]-*N*’-[2’-(4’’-(3-trifluoromethyl-3*H*-diazirin-3-yl)phenylamido)ethyl]}squaric acid diamide (4).** The squaric acid diamide **11** (152 mg, 290 µmol) was dissolved in distilled water which contained 50% TFA (9 mL). The solution was then stirred at room temperature for 3 h. After complete conversion, the solvent was removed under reduced pressure, codistilled with toluene and neutralized with basic ion exchange resin in methanol. The resin was filtered off and the solvent removed under reduced pressure. The primary amine was then dried under vacuum together with the diazirine **8** [28] (56.0 mg, 250 µmol) und HATU (114 mg, 300 µmol) for 30 min. The substances were dissolved in dry DMF (10 mL) and DIPEA (20.0 µL, 180 µmol) was added. The solution was stirred at room temperature for 16 h. Subsequently, the solvent was removed under reduced pressure and the crude product was purified by flash column chromatography (dichloromethane/methanol 8:1→4:1). The product was obtained as a colorless solid (123 mg) containing minor amounts of side products which could not be separated (crude yield 68%). ^1^H NMR (500 MHz, MeOH-*d*_4_) δ 7.96–7.92 (m, 2H, CH_aryl_-2‘,6‘), 7.39 (d, ^3^*J* = 8.8 Hz, 2H, CH_aryl_-2,6), 7.35 (d, ^3^*J* = 8.2 Hz, 2H, CH_aryl_-3‘,5‘), 7.14–7.10 (m, 2H, CH_aryl_-3,5), 5.47 (d, ^3^*J*_1,2_ = 1.8 Hz, 1H, H-1), 4.04 (dd, ^3^*J*_2,1_ = 1.8 Hz, ^3^*J*_2,3_ = 3.4 Hz, 1H, H-2), 3.93 (dd, ^3^*J*_3,2_ = 3.4 Hz, ^3^*J*_3,4_ = 9.4 Hz, H-3), 3.80 (dd, ^3^*J*_6,5_ = 2.6 Hz, ^2^*J*_6,6‘_ = 12.0 Hz, 1H, H-6), 3.78–3.73 (m, 2H, H-4, H-6‘), 3.70–3.66 (m, 2H, CH_2-ethyl_) 3.64 (ddd, ^3^*J*_5,4_ = 9.8 Hz, ^3^*J*_5,6‘_ = 4.5 Hz, ^3^*J*_5,6_ = 2.6 Hz, 1H, H-5), 3.29–3.23 (m, 2H, CH_2-ethyl_) ppm; ^13^C NMR (126 MHz, MeOH-*d*_4_, 300 K) δ 169.3, 165.6 (2C, *C*_squaric acid_), 154.6 (1C, C_aryl_-4), 137.0 (1C, C_aryl_-1‘‘), 134.6 (1C, C_aryl_-1), 133.2 (1C, C_aryl_-4’’), 129.1 (2C, CH_aryl_-2‘‘,6‘‘), 127.7 (2C, CH_aryl_-3‘‘,5‘‘), 121.5 (2C, CH_aryl_-2,6), 118.7 (2C, CH_aryl_-3,5), 100.6 (1C, C-1), 75.4 (1C, C-5), 72.4 (1C, C-3), 72.0 (1C, C-2), 60.3 (1C, C-4), 62.6 (1C, C-6), 43.8 (1C, CH_2-ethyl_), 42.1 (1C, CH_2-ethyl_) ppm; not visible owing to slow relaxation: 2 squaric acid C=O), CF_3_; ^19^F NMR (471 MHz, MeOH-*d*_4_) δ −66.84 (s) ppm; MALDI–TOF MS *m*/*z*: 620.72 [M]^+^.

***N*^α^-Boc-glycin-[*p*-(α-D-mannopyranosyloxy)]phenyl amide (7).** The aminophenyl mannoside **6** [26–27] (150 mg, 553 µmol) was dried together with *N*-Boc-glycine (64.6 mg, 369 µmol) and HATU (280 mg, 738 µmol) for 45 min under vacuum. Afterwards, this mixture was dissolved in dry DMF (8 mL), DIPEA (80.0 µL, 443 µmol) was added and the reaction mixture stirred overnight at room temperature. The solvent was removed under vacuum and purified with flash column chromatography (ethyl acetate/methanol 6:1) leading to a colorless solid (158 mg, 369 µmol, quant.); *R*_f_ = 0.15 (ethyl acetate/methanol 6:1); mp 74 °C; [α]_D_^21^ = 91.8 (*c* 0.5, MeOH); FTIR (KBr) 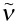: 3388, 2936, 1660, 1508, 1445, 1368, 1220, 1161, 1015, 978, 835, 556 cm^−1^; ^1^H NMR (600 MHz, MeOH-*d*_4_) δ 7.46 (d, ^3^*J* = 8.9 Hz, 2H, CH_aryl_-2,6), 7.08 (d, ^3^*J* = 8.9 Hz, 2H, CH_aryl_-3,5), 5.43 (d, ^3^*J*_1,2_ = 1.8 Hz, 1H, H-1), 3.99 (dd, ^3^*J*_2,1_ = 1.8 Hz, ^3^*J*_2,3_ = 3.4 Hz, 1H, H-2), 3.89 (dd, ^3^*J*_3,2_ = 3.4 Hz, ^3^*J*_3,4_ = 9.4 Hz, 1H, H-3), 3.84 (s, 2H, CH_2-Glycin_), 3.76 (dd, ^3^*J*_6‘,5_ = 2.4 Hz, ^2^*J*_6‘,6_ = 11.9 Hz, 1H, H-6‘), 3.75–3.69 (m, 2H, H-6, H-4), 3.61 (ddd, ^3^*J*_5,6‘_ = 2.4 Hz, ^3^*J*_5,6_ = 5.1 Hz, ^3^*J*_5,4_ = 9.4 Hz, 1H, H-5), 1.47 (s, 9H, C(CH_3_)_3_) ppm; ^13^C NMR (151 MHz, MeOH-*d*_4_) δ 170.4 (1C, C=O_glycine_), 158.6 (1C, C=O_Boc_), 154.6 (1C, C_aryl_-4), 134.1 (1C, C_aryl_-1), 122.9 (2C, CH_aryl_-2,6), 118.1 (2C, CH_aryl_-3,5), 100.5 (1C, C-1), 80.7 (1C, *C*(CH_3_)_3_), 75.4 (1C, C-5), 72.4 (1C, C-3), 72.0 (1C, C-2), 68.4 (1C, C-4), 62.7 (1C, C-6), 45.0 (1C, CH_2-glycine_), 28.7 (3C, C(*C*H_3_)_3_) ppm; MALDI–TOF MS *m*/*z*: 451.10 [M + Na]^+^; ESIMS *m*/*z*: 448.3 [M + Na]^+^, 467.3 ([M + K]^+^.

***N*-{[4-(α-D-Mannopyranosyloxy)phenyl]amido}squaric acid methyl ester (10).** To a solution of the aminophenyl mannoside **6** [26–27] (200 mg, 737 µmol) in dry methanol (15 mL), squaric acid dimethyl ester (**9**, 314 mg, 2.21 mmol) was added. The solution was stirred at room temperature for 16 h. Subsequently, the solvent was removed under reduced pressure and the crude product was purified by flash column chromatography (ethyl acetate/methanol 3:1). The product was isolated as a colorless solid (166 mg, 436 µmol, 59%); *R*_f_ = 0.43 (ethyl acetate/methanol, 2:1); mp 198 °C (decomp.); [α]_D_^21^ = +88.0 (*c* 0.4, DMSO); FTIR (KBr) 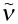: 3260, 1797, 1698, 1624, 1586, 1396, 1234, 1004, 811 cm^−1^; ^1^H NMR (200 MHz, MeOH-*d*_4_) δ 7.30 (d, ^3^*J* = 9.0 Hz, 2H, CH_aryl_-2,6), 7.12 (d, ^3^*J* = 9.0 Hz, 2H, CH_aryl_-3,5), 5.45 (d, ^3^*J*_1,2_ = 1.8 Hz, 1H, H-1), 4.44 (s, 3H, OCH_3_), 4.01 (dd, ^3^*J*_2,1_ = 1.8 Hz, ^3^*J*_2,3_ = 3.4 Hz, 1H, H-2), 3.89 (dd, ^3^*J*_3,2_ = 3.4 Hz, ^3^*J*_3,4_ = 9.4 Hz, 1H, H-3), 3.80 (dd, ^3^*J*_6‘,5_ = 2.8 Hz, ^2^*J*_6‘,6_ = 11.9 Hz, 1H, H-6‘), 3.73 (dd~t, ^3^*J*_4,3_ = ^3^*J*_4,5_ = 9.4 Hz, 1H, H-4), 3.71 (dd, ^3^*J*_6,5_ = 4.6 Hz, ^2^*J*_6,6‘_ = 11.9 Hz, 1H, H-6), 3.60 (ddd, ^3^*J*_5,6‘_ = 2.8 Hz, ^3^*J*_5,6_ = 4.6 Hz, ^3^*J*_5,4_ = 9.4 Hz, 1H, H-5) ppm; ^13^C NMR (151 MHz, MeOH-*d*_4_, 300 K) δ 193.9, 193.6 (2C, C=O_squaric acid_), 179.8, 164.9 (2C, C_squaric acid_), 154.4 (1C, C_aryl_), 132.8 (1C, C_aryl_), 122.6 (2C, CH_aryl_), 118.5 (2C, CH_aryl_), 100.5 (1C, C-1), 75.5 (1C, C-5), 72.4 (1C, C-3), 72.0 (1C, C-2), 68.4 (1C, C-4), 62.7 (1C, C-6), 59.8 (1C, OCH_3_) ppm; MALDI–TOF MS *m*/*z*: 404.46 [M + Na]^+^.

**{*N*-[4-(α-D-Mannopyranosyloxy)phenyl]-*N*’-[(2‘-*tert*-butyloxycarbonylamido)ethyl]}squaric acid diamide (11).** The squaric acid monoamide **10** (90.0 mg, 240 µmol) was dissolved in dry methanol (5 mL). Afterwards, *N*-Boc-ethylendiamine (46.0 mg, 465 µmol) and triethylamine (140 µL) were added. The reaction solution was stirred at room temperature for 15 h. The solution was neutralized with an acidic ion exchange resin (Amberlyst-A21), filtered and concentrated under reduced pressure. The crude product was purified by column chromatography (ethyl acetate/methanol 3:1), leading to a colorless solid (114 mg, 220 µmol, 92%); *R*_f_ = 0.44 (ethyl acetate/methanol 3:1); mp 176–185 °C (decomp.); [α]_D_^25^ = +72.1 (*c* 0.3, CH_3_OH); FTIR (KBr) 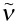: 3359, 2970, 2933, 1798, 1689, 1650, 1609, 1550, 1514, 1454, 1285, 1118, 1021, 918, 881, 826, 765 cm^−1^; ^1^H NMR (500 MHz, MeOH-*d*_4_) δ 7.34 (d, ^3^*J* = 8.8 Hz, 2H, CH_aryl_-2,6), 7.13 (d, ^3^*J* = 8.8 Hz, 2H, CH_aryl_-3,5), 5.44 (d, ^3^*J*_1,2_ = 1.9 Hz, 1H, H-1), 4.00 (dd, ^3^*J*_2,1_ = 1.9 Hz, ^3^*J*_2,3_ = 3.5 Hz, 1H, H-2), 3.89 (dd, ^3^*J*_3,2_ = 3.5 Hz, ^3^*J*_3,4_ = 9.4 Hz, 1H, H-3), 3.78 (dd, ^3^*J*_6,5_ = 2.5 Hz, ^2^*J*_6,6‘_ = 11.9 Hz, 1H, H-6), 3.75–3.69 (m, 2H, Boc-NHCH_2_C*H*_2_), 3.72 (dd~t, ^3^*J*_4,3_ = ^3^*J*_4,5_ = 9.4 Hz, 1H, H-4), 3.71 (dd, ^3^*J*_6‘,5_ = 5.4 Hz, ^2^*J*_6‘,6_ = 11.9 Hz, 1H, H-6‘), 3.62 (ddd, ^3^*J*_5,6_ = 2.5 Hz, ^3^*J*_5,6‘_ = 5.4 Hz, ^3^*J*_5,4_ = 9.4 Hz, 1H, H-5), 3.29 (m_c_, 2H, Boc-NHC*H*_2_CH_2_), 1.40 (s, 9H, C(CH_3_)_3_) ppm; ^13^C NMR (126 MHz, MeOH-*d*_4_, 300 K) δ 185.0, 182.6 (2C, C=O_squaric acid_), 170.7, 165.5 (2C, C_squaric acid_), 154.7 (1C, C_aryl_-4), 134.6 (1C, C_aryl_-1), 121.7 (2C, CH_aryl_-2,6), 118.8 (2C, CH_aryl_-3,5), 100.6 (1C, C-1), 80.4 (1C, *C*(CH_3_)_3_), 75.4 (1C, C-5), 72.4 (1C, C-3), 72.0 (1C, C-2), 68.4 (1C, C-4), 62.7 (1C, C-6), 45.3 (1C, Boc-NHCH_2_*C*H_2_), 42.4 (1C, Boc-NH*C*H_2_CH_2_), 28.7 (3C, C(*C*H_3_)_3_) ppm; MALDI–TOF MS *m*/*z*: 532.16 [M + Na]^+^, 548.14 [M + K]^+^; ESIMS *m*/*z*: 532.3 [M + Na]^+^.

## Supporting Information

File 1 NMR spectra of the synthetic compounds **3**, **4**, **7**, and **11**, docking results obtained with **3** and **4**, and MS and MS/MS spectra of labeling experiments.
