# Supplementary material for: Diazirine-functionalized mannosides for photoaffinity labeling: trouble with FimH
Source: Beilstein J Org Chem. 2018 Jul 24;14:1890–900. doi: 10.3762/bjoc.14.163 (PMC6071696; doi:10.3762/bjoc.14.163)
Supplement: File 1 — NMR spectra of the synthetic compounds 3, 4, 7, and 11, docking results obtained with 3 and 4, and MS and MS/MS spectra of labeling experiments. [file Beilstein_J_Org_Chem-14-1890-s001.pdf]

Supporting Information

for

Diazirine-functionalized mannosides for photoaffinity labeling: trouble with FimH

Femke Beiroth[1], Tomas Koudelka[2], Thorsten Overath[2], Stefan D. Knight[3], Andreas Tholey[2] and Thisbe K. Lindhorst*[,1]

Address: [1]Otto Diels Institute of Organic Chemistry, Christiana Albertina University of Kiel, Otto-Hahn-Platz 3/4, 24118 Kiel, Germany, [2]Systematic Proteomics & Bioanalytics, Institute for Experimental Medicine, Christiana Albertina University of Kiel, Niemannsweg 11, D-24105 Kiel, Germany and [3]Department of Cell and Molecular Biology, Uppsala University, Uppsala Biomedical Centre, P.O. Box 596, S-751 24 Uppsala, Sweden

Email: Thisbe K. Lindhorst* - tklind@oc.uni-kiel.de

* Corresponding author

NMR spectra of the synthetic compounds 3, 4, 7, and 11, docking results obtained with 3 and 4, and MS and MS/MS spectra of labeling experiments

Contents

1. NMR spectra of synthetic compounds
2. Computational docking studies with ligands **3** and **4**
3. MS analysis of labeling experiments
4. References

1. NMR spectra of synthetic compounds

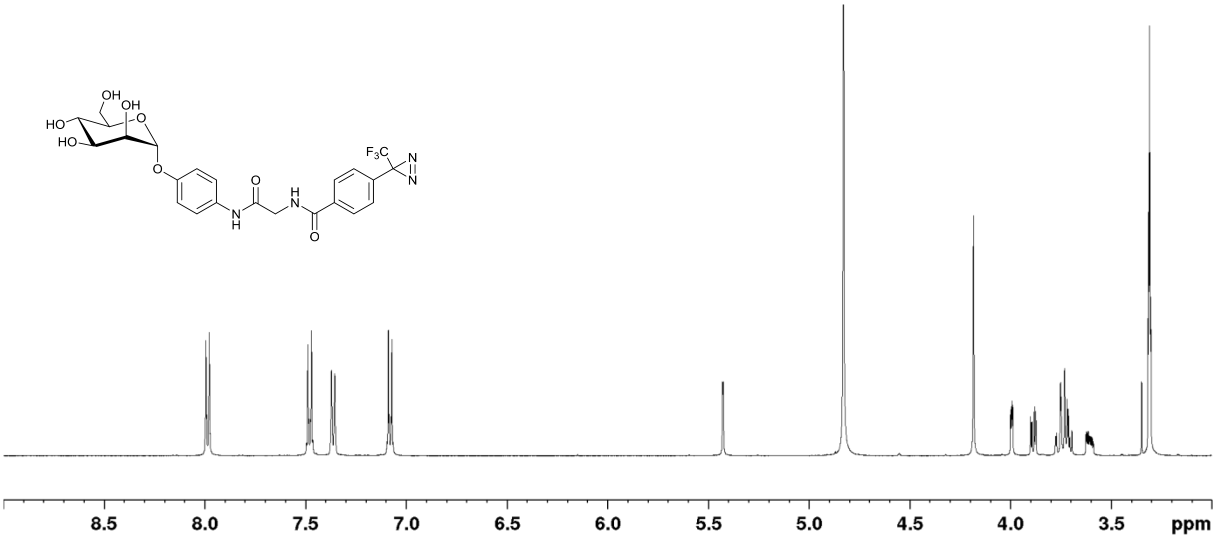

Figure S1: ^{1}H NMR spectrum of compound **3** in MeOH-d_4 (500 MHz).

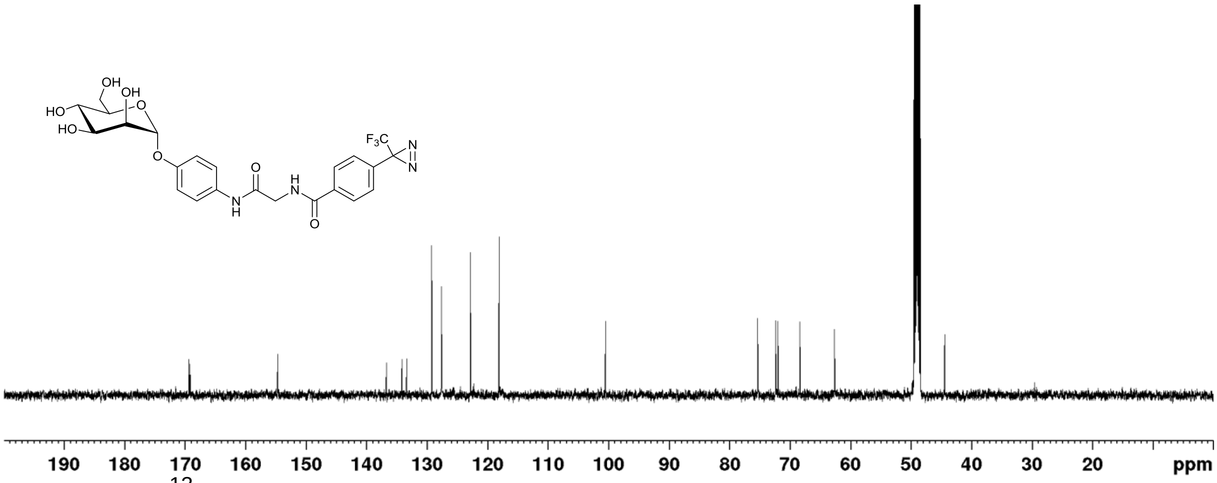

Figure S2: ^{13}C NMR spectrum of compound **3** in MeOH-d_4 (126 MHz).

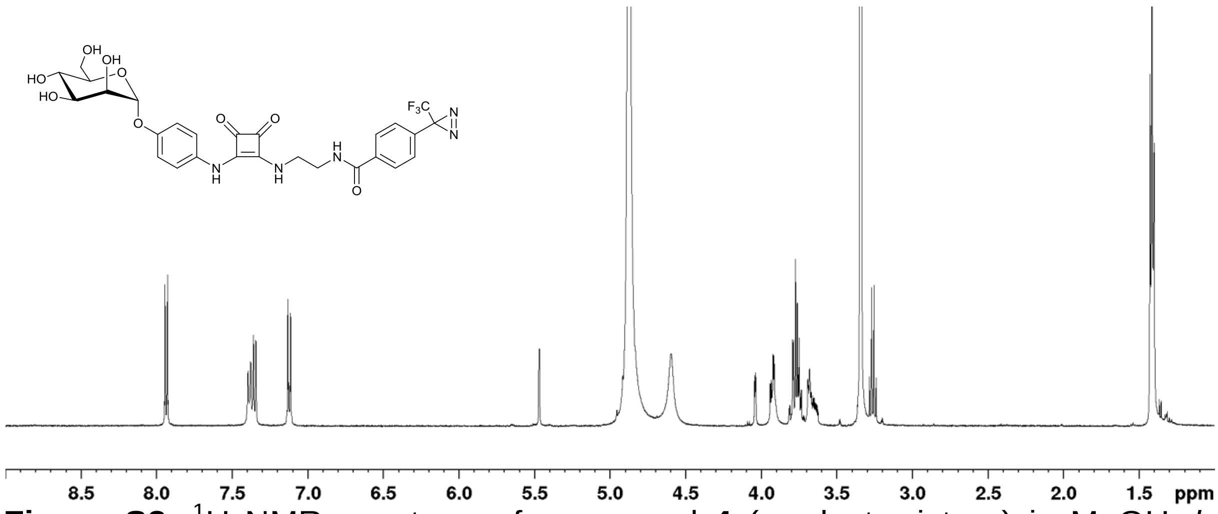

Figure S3: ^{1}H NMR spectrum of compound **4** (product mixture) in MeOH-d_4 (500 MHz).

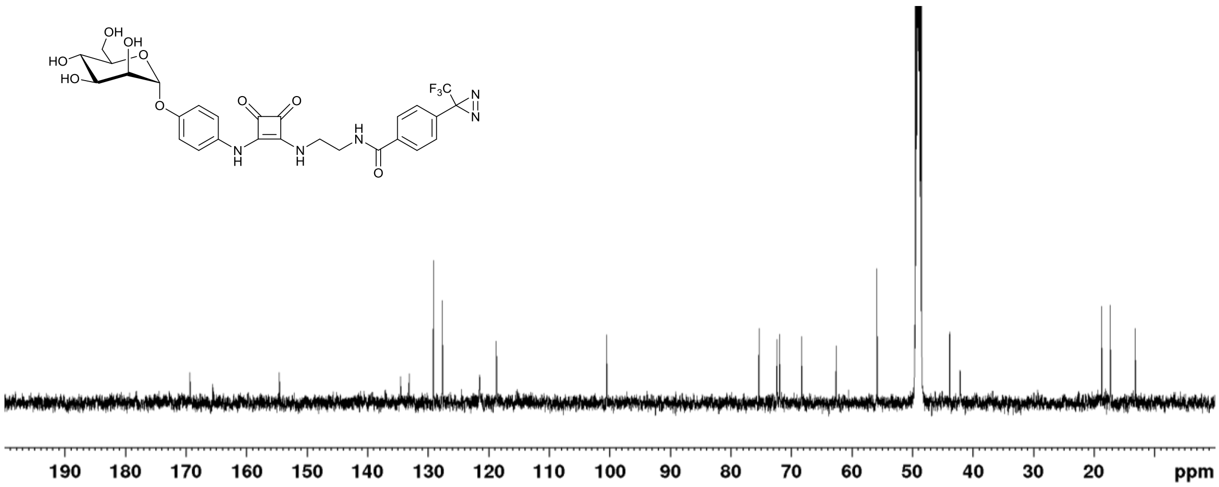

Figure S4: ^{13}C NMR spectrum of compound **4** (product mixture) in MeOH-d_4 (126 MHz).

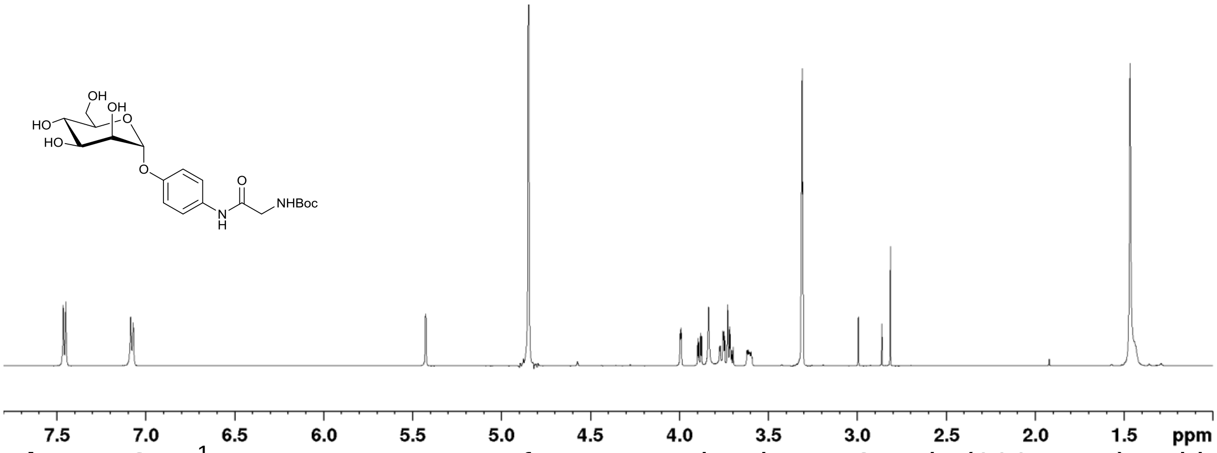

Figure S5: ^{1}H NMR spectrum of compound **7** in MeOH-d_4 (600 MHz); with residual DMF (solvent).

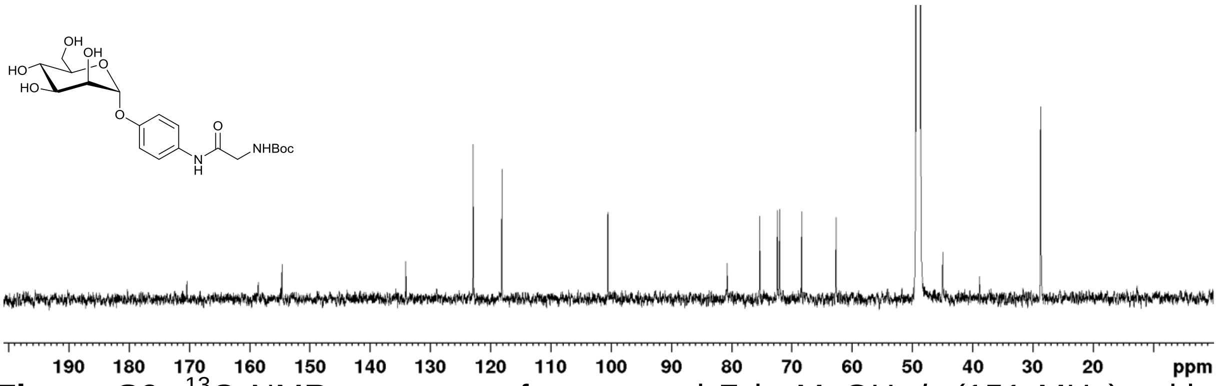

Figure S6: ^{13}C NMR spectrum of compound **7** in MeOH-d_4 (151 MHz); with residual DMF (solvent).

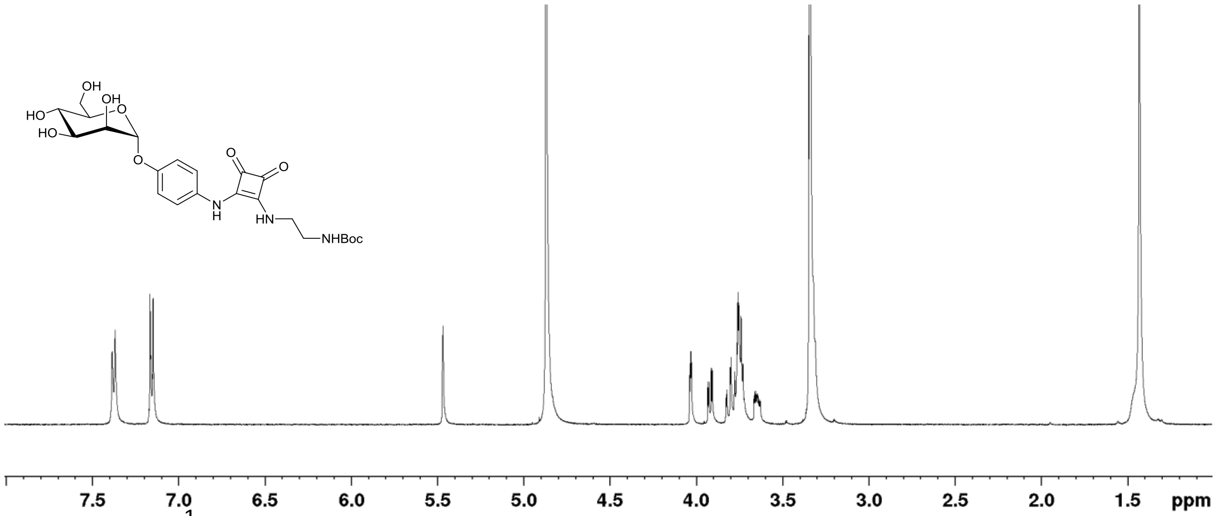

Figure S7: ^{1}H NMR spectrum of compound **11** in MeOH-d_4 (500 MHz).

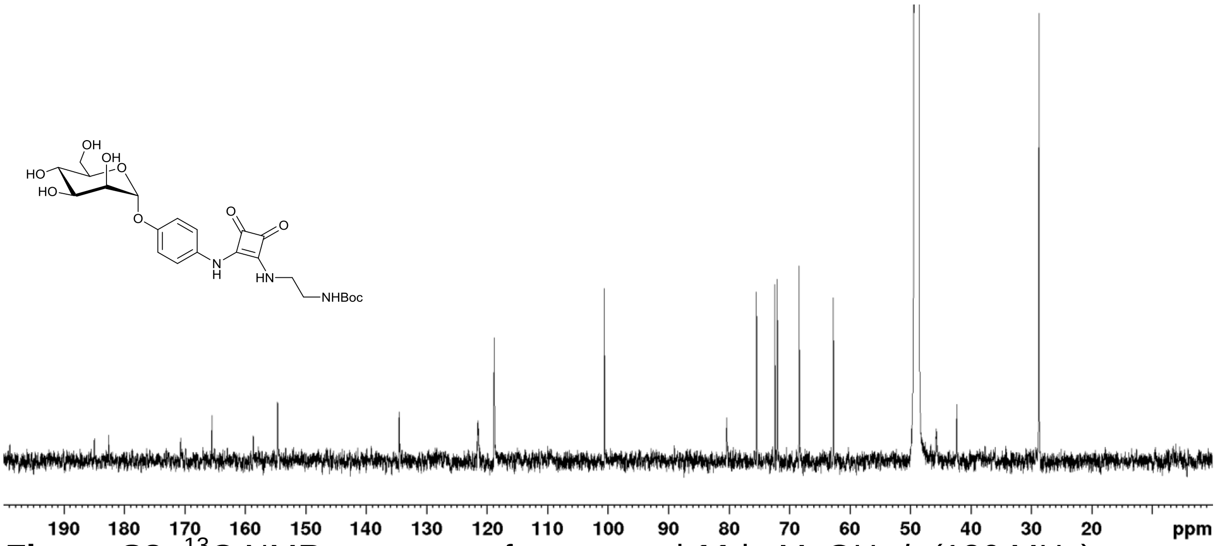

Figure S8: ^{13}C NMR spectrum of compound **11** in MeOH-d_4 (126 MHz).

2. Computational docking studies with ligands **3** and **4**

Computer-aided docking studies were performed with FlexX flexible docking [1-3] and consensus scoring [4,5] as implemented in Sybyl 6.9 [6]. For the docking, 30 conformations of a minimized ligand structure of each ligand were generated and docked into two different crystal structures [7,8] of the bacterial lectin FimH (1KLF: “open gate”; 1UWF: “closed gate” structure) that were held rigid during the calculation process. Minimization and docking were performed with the Tripos force field and Gasteiger–Hückel charges. For each ligand conformation, a FlexX scoring value was obtained that correlates with the affinity of the ligand to the lectin binding domain. The results are listed in Tables S1–4.

Table S1: Scoring values obtained from docking ligand **3** into the closed gate structure of FimH.

No.	Total Score	Match-Score	Lipo-Score	Ambig-Score	Clash-Score	Rot-Score	RMS-Value	Simil. Index	#Match	Avg. Volume	Max. Volume	Frag. No.
1	-36.173	-40.551	-9.784	-8.910	2.271	15.400	0.000	-1.000	21	0.058	0.470	1
2	-36.144	-40.551	-9.678	-8.986	2.271	15.400	1.368	-1.000	21	0.058	0.470	1
3	-36.083	-40.551	-9.793	-9.218	2.679	15.400	1.322	-1.000	21	0.069	0.470	1
4	-35.976	-39.014	-10.173	-8.609	1.021	15.400	1.548	-1.000	23	0.021	0.275	1
5	-35.938	-39.014	-10.135	-8.609	1.021	15.400	1.573	-1.000	23	0.021	0.275	1
6	-35.794	-40.366	-9.582	-8.534	1.887	15.400	2.625	-1.000	23	0.047	0.454	1
7	-35.794	-40.366	-9.582	-8.534	1.887	15.400	2.845	-1.000	23	0.047	0.454	1
8	-35.794	-40.366	-9.582	-8.534	1.887	15.400	2.818	-1.000	23	0.047	0.454	1
9	-35.794	-40.366	-9.582	-8.534	1.887	15.400	2.897	-1.000	23	0.047	0.454	1
10	-35.794	-40.366	-9.582	-8.534	1.887	15.400	2.895	-1.000	23	0.047	0.454	1
11	-35.715	-39.014	-9.912	-8.609	1.021	15.400	1.709	-1.000	23	0.021	0.275	1
12	-35.672	-40.551	-9.501	-8.846	2.426	15.400	0.439	-1.000	21	0.061	0.470	1
13	-35.652	-40.477	-9.382	-9.437	2.845	15.400	1.379	-1.000	21	0.098	1.788	1
14	-35.642	-39.014	-9.840	-8.609	1.021	15.400	1.620	-1.000	23	0.021	0.275	1
15	-35.545	-39.014	-9.742	-8.609	1.021	15.400	1.777	-1.000	23	0.021	0.275	1
16	-35.545	-39.014	-9.742	-8.609	1.021	15.400	1.776	-1.000	23	0.021	0.275	1
17	-35.358	-40.514	-9.559	-8.329	2.244	15.400	2.865	-1.000	23	0.057	0.548	1
18	-35.358	-40.514	-9.559	-8.329	2.244	15.400	2.868	-1.000	23	0.057	0.548	1
19	-35.358	-40.514	-9.559	-8.329	2.244	15.400	2.727	-1.000	23	0.057	0.548	1
20	-35.358	-40.514	-9.559	-8.329	2.244	15.400	2.623	-1.000	23	0.057	0.548	1
21	-35.358	-40.514	-9.559	-8.329	2.244	15.400	2.682	-1.000	23	0.057	0.548	1
22	-35.358	-40.514	-9.559	-8.329	2.244	15.400	2.642	-1.000	23	0.057	0.548	1
23	-35.358	-40.514	-9.559	-8.329	2.244	15.400	2.757	-1.000	23	0.057	0.548	1
24	-35.292	-40.477	-9.388	-9.071	2.845	15.400	0.446	-1.000	21	0.098	1.788	1
25	-35.267	-40.551	-9.382	-8.817	2.682	15.400	1.152	-1.000	21	0.074	0.612	1
26	-35.098	-40.779	-9.353	-8.310	2.545	15.400	2.548	-1.000	23	0.069	0.583	1
27	-35.098	-40.779	-9.353	-8.310	2.545	15.400	2.623	-1.000	23	0.069	0.583	1
28	-35.098	-40.779	-9.353	-8.310	2.545	15.400	2.859	-1.000	23	0.069	0.583	1
29	-35.098	-40.779	-9.353	-8.310	2.545	15.400	2.810	-1.000	23	0.069	0.583	1
30	-35.098	-40.779	-9.353	-8.310	2.545	15.400	2.815	-1.000	23	0.069	0.583	1

Table S2: Scoring values obtained from docking ligand **3** into the open gate structure of FimH.

No.	Total Score	Match-Score	Lipo-Score	Ambig-Score	Clash-Score	Rot-Score	RMS-Value	Simil. Index	#Match	Avg. Volume	Max. Volume	Frag. No.
1	-34.647	-39.761	-9.693	-8.640	2.647	15.400	0.000	-1.000	26	0.112	1.602	1
2	-33.226	-37.598	-8.577	-9.678	1.827	15.400	6.162	-1.000	26	0.046	0.569	1
3	-33.176	-37.598	-8.527	-9.678	1.827	15.400	6.248	-1.000	26	0.046	0.569	1
4	-33.020	-37.598	-8.315	-9.735	1.827	15.400	5.972	-1.000	26	0.046	0.569	1
5	-32.964	-37.598	-8.315	-9.678	1.827	15.400	6.109	-1.000	26	0.046	0.569	1
6	-32.964	-37.598	-8.315	-9.678	1.827	15.400	5.951	-1.000	26	0.046	0.569	1
7	-32.764	-36.292	-9.597	-9.771	2.096	15.400	1.342	-1.000	24	0.047	0.451	1
8	-32.572	-38.511	-7.913	-9.298	2.350	15.400	5.782	-1.000	27	0.075	1.125	1
9	-32.563	-38.511	-7.913	-9.289	2.350	15.400	5.890	-1.000	27	0.075	1.125	1
10	-32.531	-38.511	-7.927	-9.242	2.350	15.400	6.065	-1.000	27	0.075	1.125	1
11	-32.516	-38.511	-7.913	-9.242	2.350	15.400	5.752	-1.000	27	0.075	1.125	1
12	-32.516	-38.511	-7.913	-9.242	2.350	15.400	5.832	-1.000	27	0.075	1.125	1
13	-32.516	-38.511	-7.913	-9.242	2.350	15.400	6.056	-1.000	27	0.075	1.125	1
14	-32.386	-36.675	-9.099	-9.459	2.047	15.400	1.709	-1.000	25	0.138	2.482	1
15	-32.294	-39.780	-8.495	-8.524	3.704	15.400	0.684	-1.000	26	0.172	1.602	1
16	-32.266	-38.085	-7.311	-9.092	1.421	15.400	5.614	-1.000	26	0.036	0.453	1
17	-32.266	-38.085	-7.311	-9.092	1.421	15.400	5.530	-1.000	26	0.036	0.453	1
18	-32.266	-38.085	-7.311	-9.092	1.421	15.400	5.358	-1.000	26	0.036	0.453	1
19	-32.266	-38.085	-7.311	-9.092	1.421	15.400	5.282	-1.000	26	0.036	0.453	1
20	-32.266	-38.085	-7.311	-9.092	1.421	15.400	5.552	-1.000	26	0.036	0.453	1
21	-31.942	-34.442	-10.266	-10.007	1.972	15.400	1.054	-1.000	26	0.094	1.148	1
22	-31.858	-38.086	-7.077	-8.915	1.420	15.400	5.747	-1.000	26	0.036	0.449	1
23	-31.858	-38.086	-7.077	-8.915	1.420	15.400	5.409	-1.000	26	0.036	0.449	1
24	-31.858	-38.086	-7.077	-8.915	1.420	15.400	5.768	-1.000	26	0.036	0.449	1
25	-31.858	-38.086	-7.077	-8.915	1.420	15.400	5.682	-1.000	26	0.036	0.449	1
26	-31.858	-38.086	-7.077	-8.915	1.420	15.400	5.444	-1.000	26	0.036	0.449	1
27	-31.699	-37.118	-9.878	-9.024	3.520	15.400	1.395	-1.000	27	0.217	2.313	1
28	-31.509	-37.129	-9.331	-8.880	3.031	15.400	1.520	-1.000	26	0.174	1.931	1
29	-31.498	-36.154	-9.496	-9.574	2.926	15.400	2.075	-1.000	24	0.140	1.522	1
30	-31.495	-36.389	-10.392	-8.883	3.368	15.400	0.738	-1.000	23	0.131	2.344	1

Table S3: Scoring values obtained from docking ligand **4** into the closed gate structure of FimH.

No.	Total Score	Match-Score	Lipo-Score	Ambig-Score	Clash-Score	Rot-Score	RMS-Value	Simil. Index	#Match	Avg. Volume	Max. Volume	Frag No.
1	-34.007	-41.701	-9.500	-11.718	2.512	21.000	0.000	-1.000	19	0.065	0.693	1
2	-33.392	-43.004	-9.241	-9.394	1.847	21.000	2.252	-1.000	19	0.051	0.934	1
3	-33.019	-42.402	-8.061	-11.370	2.414	21.000	0.643	-1.000	20	0.070	0.600	1
4	-32.822	-41.946	-9.149	-9.281	1.155	21.000	2.192	-1.000	18	0.023	0.254	1
5	-32.773	-42.582	-8.053	-10.959	2.421	21.000	0.934	-1.000	19	0.076	1.255	1
6	-31.643	-41.302	-9.348	-9.527	2.134	21.000	1.792	-1.000	19	0.055	0.932	1
7	-31.556	-40.579	-9.847	-8.930	1.400	21.000	2.362	-1.000	19	0.042	1.092	1
8	-31.497	-40.667	-9.180	-9.497	1.447	21.000	1.739	-1.000	17	0.028	0.293	1
9	-31.380	-43.527	-8.169	-9.648	3.565	21.000	8.921	-1.000	19	0.147	2.043	1
10	-31.221	-41.305	-12.648	-11.694	8.026	21.000	1.242	-1.000	19	0.295	2.368	1
11	-31.127	-40.168	-8.793	-9.959	1.394	21.000	1.997	-1.000	15	0.032	0.269	1
12	-31.080	-40.830	-7.940	-11.088	2.379	21.000	9.858	-1.000	17	0.067	0.769	1
13	-30.914	-41.335	-11.747	-9.777	5.544	21.000	2.420	-1.000	18	0.218	1.922	1
14	-30.838	-42.753	-7.919	-10.563	3.997	21.000	0.971	-1.000	19	0.152	2.362	1
15	-30.742	-42.753	-7.919	-10.445	3.975	21.000	0.559	-1.000	19	0.151	2.362	1
16	-30.580	-41.441	-11.422	-10.337	6.220	21.000	9.605	-1.000	17	0.214	2.183	1
17	-30.452	-40.466	-8.120	-10.120	1.854	21.000	2.027	-1.000	17	0.072	1.525	1
18	-30.439	-40.507	-8.862	-9.655	2.186	21.000	1.829	-1.000	15	0.050	0.326	1
19	-30.401	-39.549	-9.470	-10.831	3.048	21.000	1.383	-1.000	20	0.085	1.006	1
20	-30.351	-39.137	-9.607	-8.655	0.649	21.000	2.306	-1.000	18	0.013	0.190	1
21	-30.299	-40.042	-8.945	-9.215	1.503	21.000	2.346	-1.000	15	0.042	0.675	1
22	-30.095	-39.784	-7.925	-11.212	2.425	21.000	1.681	-1.000	16	0.056	0.490	1
23	-30.073	-40.343	-8.472	-10.561	2.903	21.000	9.902	-1.000	17	0.092	1.028	1
24	-30.044	-41.035	-10.826	-11.505	6.923	21.000	1.386	-1.000	19	0.294	2.368	1
25	-29.830	-39.355	-8.958	-11.010	3.094	21.000	1.331	-1.000	19	0.083	0.919	1
26	-29.585	-40.752	-8.981	-9.494	3.242	21.000	2.651	-1.000	19	0.116	1.818	1
27	-29.465	-40.462	-8.133	-11.101	3.831	21.000	1.766	-1.000	17	0.126	1.761	1
28	-29.408	-40.683	-8.163	-9.645	2.683	21.000	1.817	-1.000	16	0.098	1.026	1
29	-29.331	-38.858	-8.727	-10.216	2.069	21.000	2.206	-1.000	15	0.086	1.358	1
30	-29.269	-39.784	-7.624	-10.411	2.150	21.000	1.710	-1.000	16	0.050	0.490	1

Table S4: Scoring values obtained from docking ligand **4** into the open gate structure of FimH.

No.	Total Score	Match-Score	Lipo-Score	Ambig-Score	Clash-Score	Rot-Score	RMS-Value	Simil. Index	#Match	Avg. Volume	Max. Volume	Frag No.
1	-28.588	-35.622	-11.769	-10.314	2.718	21.000	0.000	-1.000	24	0.104	2.398	1
2	-28.194	-36.336	-12.283	-10.873	4.898	21.000	7.054	-1.000	21	0.165	1.704	1
3	-27.964	-34.564	-12.975	-12.798	5.973	21.000	7.108	-1.000	20	0.240	2.182	1
4	-27.373	-35.339	-11.594	-9.698	2.857	21.000	6.787	-1.000	20	0.113	2.366	1
5	-27.271	-37.125	-10.907	-9.612	3.973	21.000	7.037	-1.000	23	0.155	2.202	1
6	-27.095	-37.125	-10.918	-9.426	3.973	21.000	6.990	-1.000	23	0.155	2.202	1
7	-27.010	-33.798	-12.394	-9.703	2.484	21.000	5.265	-1.000	22	0.118	2.130	1
8	-26.857	-34.939	-10.944	-10.170	2.797	21.000	6.713	-1.000	24	0.109	2.296	1
9	-26.706	-36.674	-10.570	-9.600	3.739	21.000	7.027	-1.000	23	0.150	2.202	1
10	-26.566	-34.820	-13.214	-10.866	5.934	21.000	7.143	-1.000	22	0.225	2.258	1
11	-26.535	-33.429	-11.574	-10.230	2.298	21.000	6.596	-1.000	23	0.091	2.235	1
12	-26.468	-33.487	-11.410	-10.230	2.258	21.000	6.590	-1.000	22	0.091	2.244	1
13	-26.455	-36.674	-10.592	-9.328	3.739	21.000	7.100	-1.000	23	0.150	2.202	1
14	-26.308	-36.674	-10.422	-9.351	3.739	21.000	6.942	-1.000	23	0.150	2.202	1
15	-26.248	-33.203	-11.586	-10.671	2.811	21.000	6.149	-1.000	22	0.102	2.317	1
16	-26.245	-33.257	-11.898	-10.454	2.964	21.000	0.883	-1.000	22	0.109	2.398	1
17	-26.200	-37.125	-10.412	-9.035	3.973	21.000	6.975	-1.000	23	0.155	2.202	1
18	-26.194	-37.125	-10.412	-9.030	3.973	21.000	7.222	-1.000	23	0.155	2.202	1
19	-26.194	-37.125	-10.412	-9.030	3.973	21.000	7.210	-1.000	23	0.155	2.202	1
20	-26.147	-32.708	-13.612	-9.562	3.335	21.000	4.843	-1.000	21	0.121	2.130	1
21	-26.114	-35.216	-12.093	-10.933	5.728	21.000	6.352	-1.000	21	0.199	2.225	1
22	-25.991	-33.018	-11.516	-10.771	2.914	21.000	6.132	-1.000	21	0.103	2.307	1
23	-25.822	-36.674	-10.252	-9.035	3.739	21.000	7.218	-1.000	23	0.150	2.202	1
24	-25.822	-36.674	-10.252	-9.035	3.739	21.000	7.193	-1.000	23	0.150	2.202	1
25	-25.822	-36.674	-10.252	-9.035	3.739	21.000	7.003	-1.000	23	0.150	2.202	1
26	-25.810	-33.429	-11.219	-9.859	2.298	21.000	6.384	-1.000	23	0.091	2.235	1
27	-25.794	-33.203	-11.030	-10.420	2.458	21.000	6.406	-1.000	22	0.095	2.317	1

28	- 25.777	- 33.490	-11.198	-10.465	2.976	21.000	6.620	-1.000	16	0.122	2.187	1
29	- 25.777	- 33.490	-11.198	-10.465	2.976	21.000	6.556	-1.000	16	0.122	2.187	1
30	- 25.777	- 33.490	-11.198	-10.465	2.976	21.000	6.887	-1.000	16	0.122	2.187	1

3. MS analysis of labeling experiments

To test carbene formation, mannoside **3** was irradiated at 345 nm in DMSO and with 4-hydroxybenzyl alcohol in 1:1 acetonitrile-water as well as in 1:1 DMSO/water mixtures. Mass-spectrometric analysis indicated carbene formation and the desired crosslinked product together with insertion into water in both cases, see Table S5.

Table S5: Scoring values obtained from docking ligand **4** into the open gate structure of FimH.

Irradiated compound(s)	Solvent	Detected products
**3**	DMSO	Carbene
**3** + 4-Hydroxybenzyl alcohol (HBA)	DMSO/water, 1:1	Insertion products<br>(**3**-N_2) + HBA<br>(**3**-N_2) + H_2O
**3** + 4-Hydroxybenzyl alcohol (HBA)	Acetontrile/water, 1:1	Insertion products<br>(**3**-N_2) + HBA<br>(**3**-N_2) + H_2O

Labeling experiments with six different peptides were performed as described in the main manuscript. The corresponding MS and MS/MS spectra are shown in Figures S9–S23.

For MS/MS analysis three different fragmentation principles were applied: electron transfer dissociation (ETD), collision induced dissociation (CID) and higher-energy collisional dissociation (HCD).

Employed model peptides: ILMEHIHKL (M2), YLLPAIVHI (M3), EIAMATVTALR (M7), ETIGEILKK (M8), EGHIARNCRA (T3) RPQYAEASWNAR (S17).

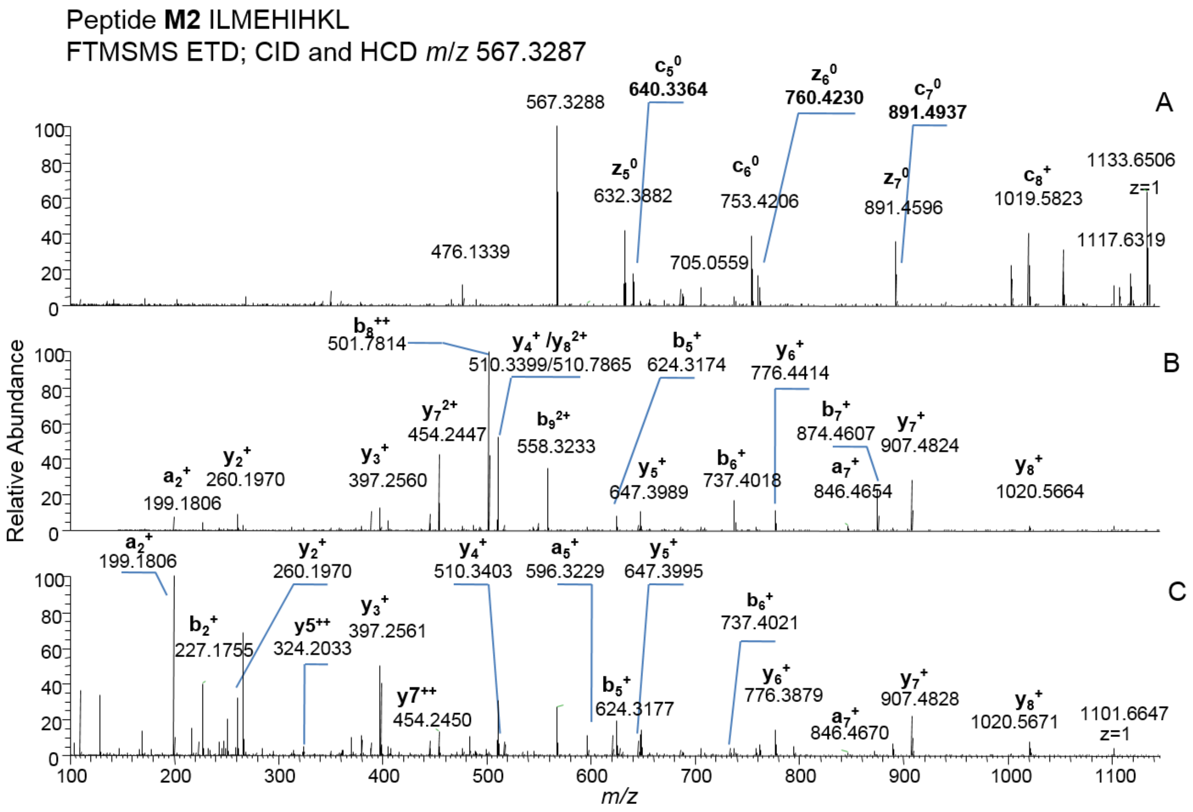

Figure S9: ESIMS/MS spectra of peptide M2. (A): ETD, (B): CID, (C): HCD.

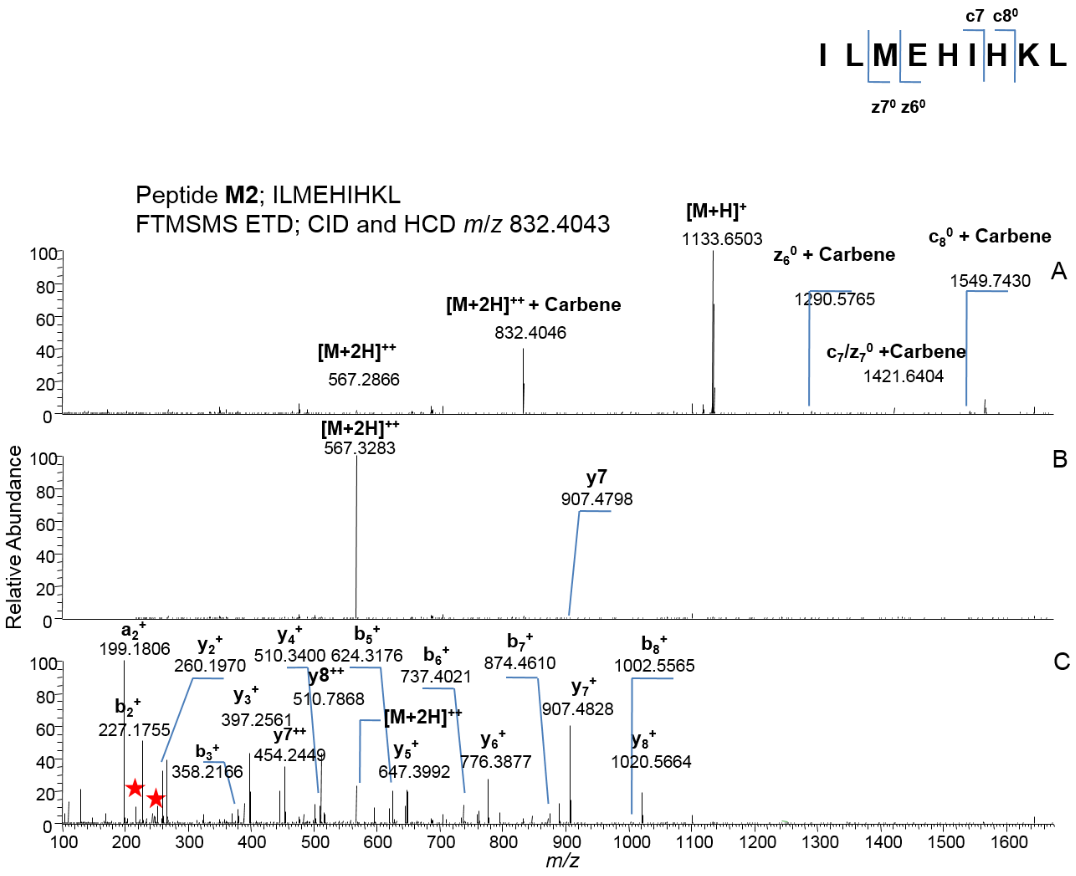

Figure S10: ESIMS/MS spectra of peptide M2 after labeling with **3**. (A): ETD, (B): CID, (C): HCD. Red stars indicate signals deriving from fragmentation of carbohydrate moieties of **3**.

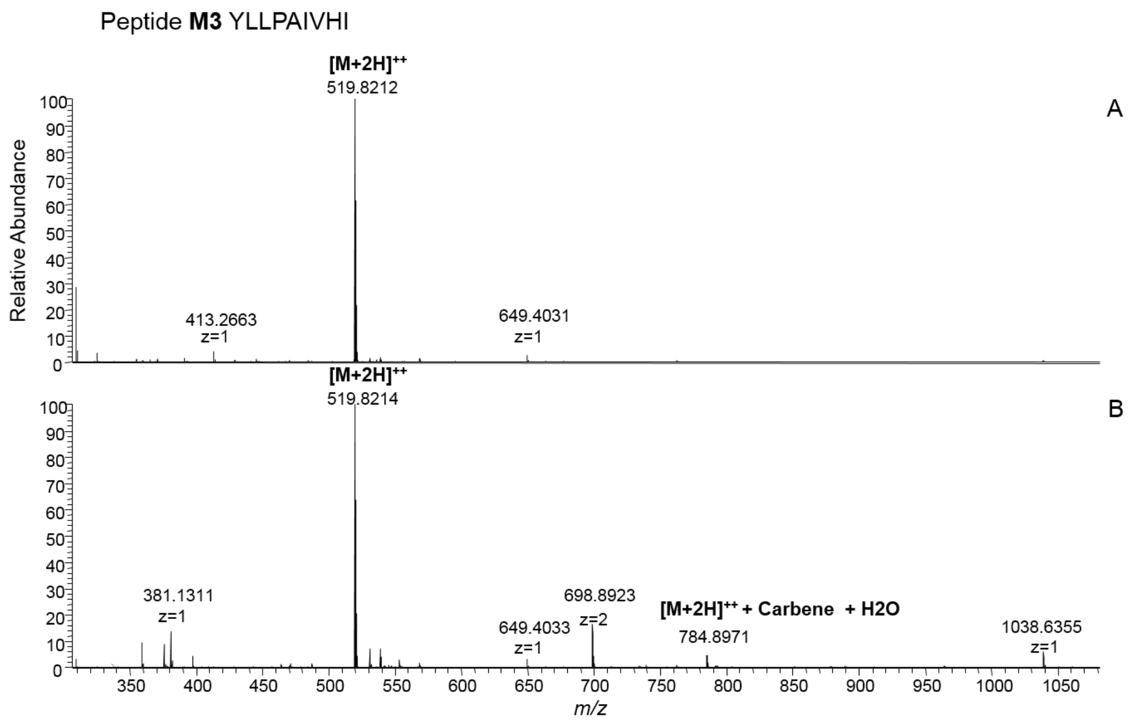

Figure S11: ESIMS spectra of peptide M3 before (A) and after (B) labeling with **3**.

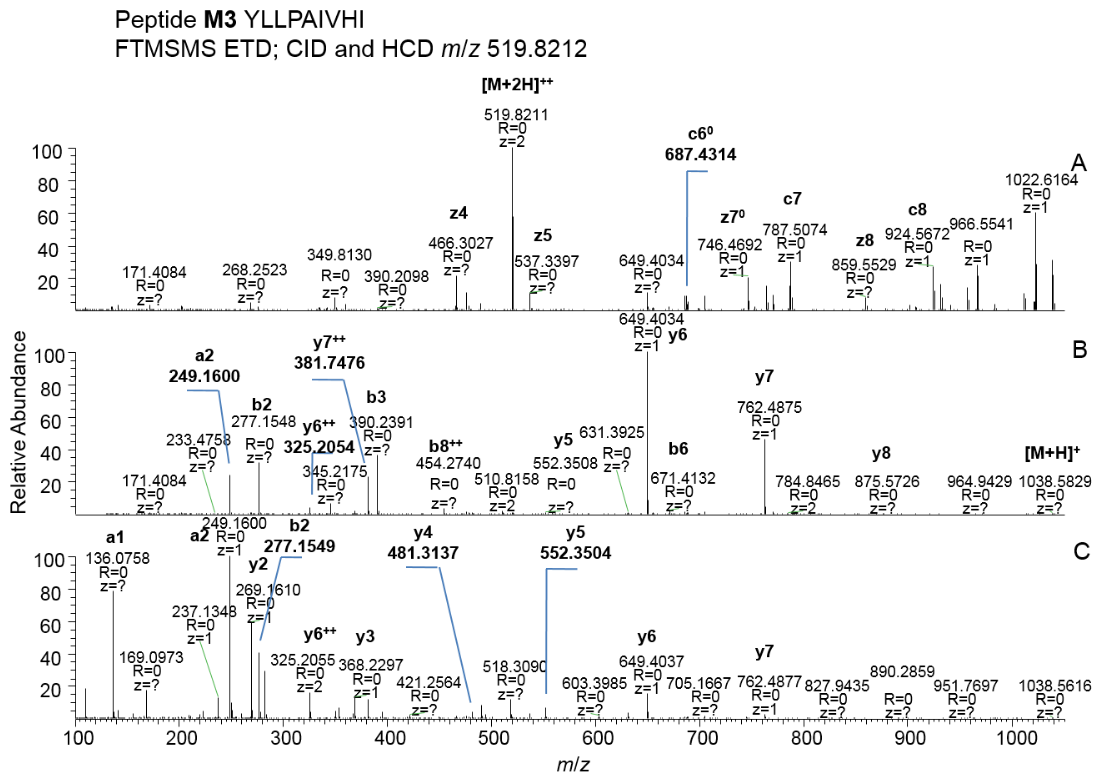

Figure S12: ESIMS/MS spectra of peptide M3. (A): ETD, (B): CID, (C): HCD.

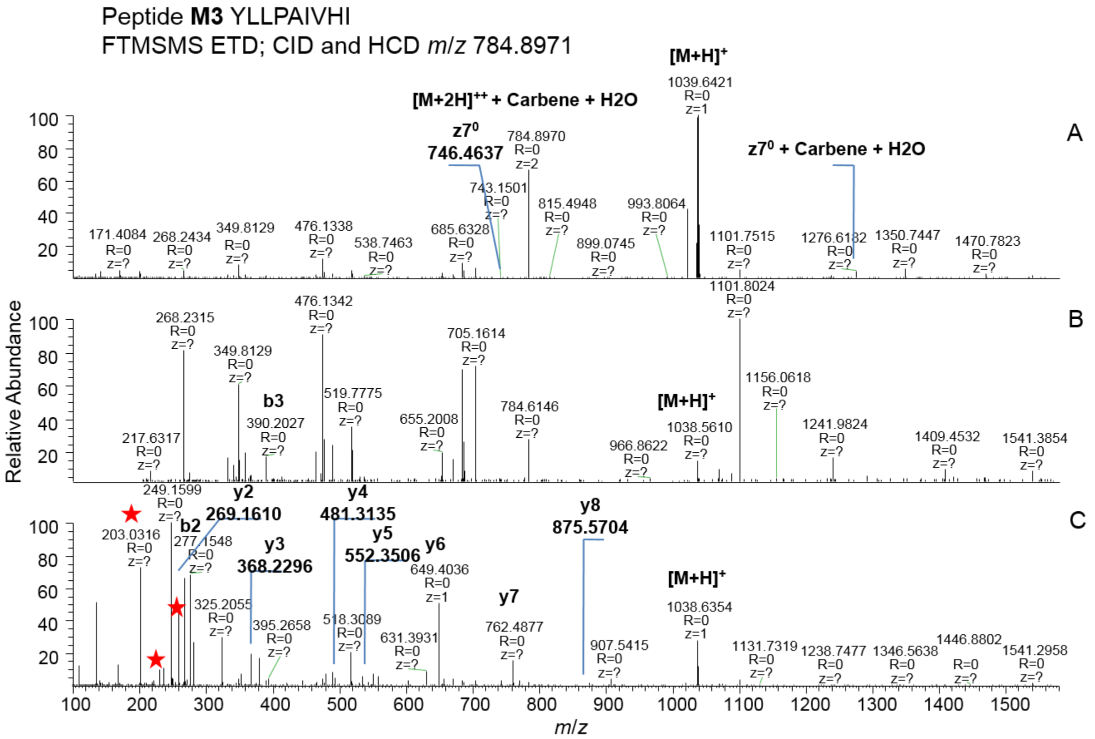

Figure S13: ESIMS/MS spectra of peptide M3 after labeling with **3**. (A): ETD, (B): CID, (C): HCD. Red stars indicate signals deriving from fragmentation of carbohydrate moieties of **3**.

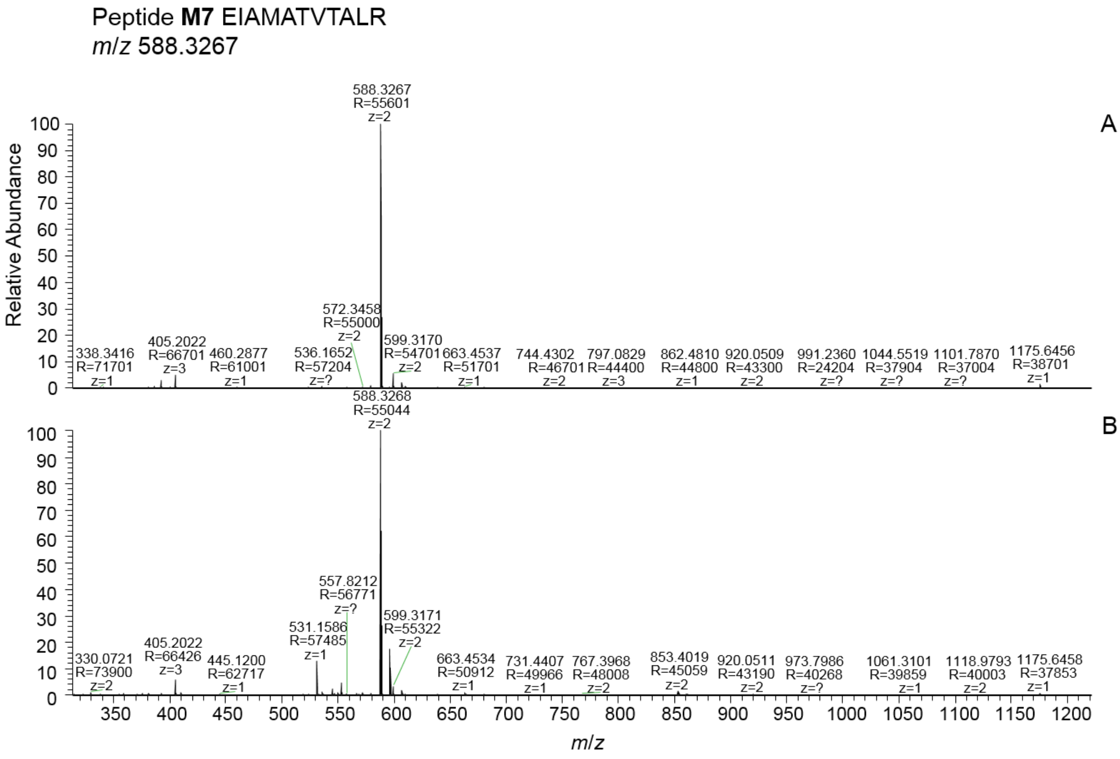

Figure S14: ESIMS spectra of peptide M7 before (A) and after (B) labeling with **3**.

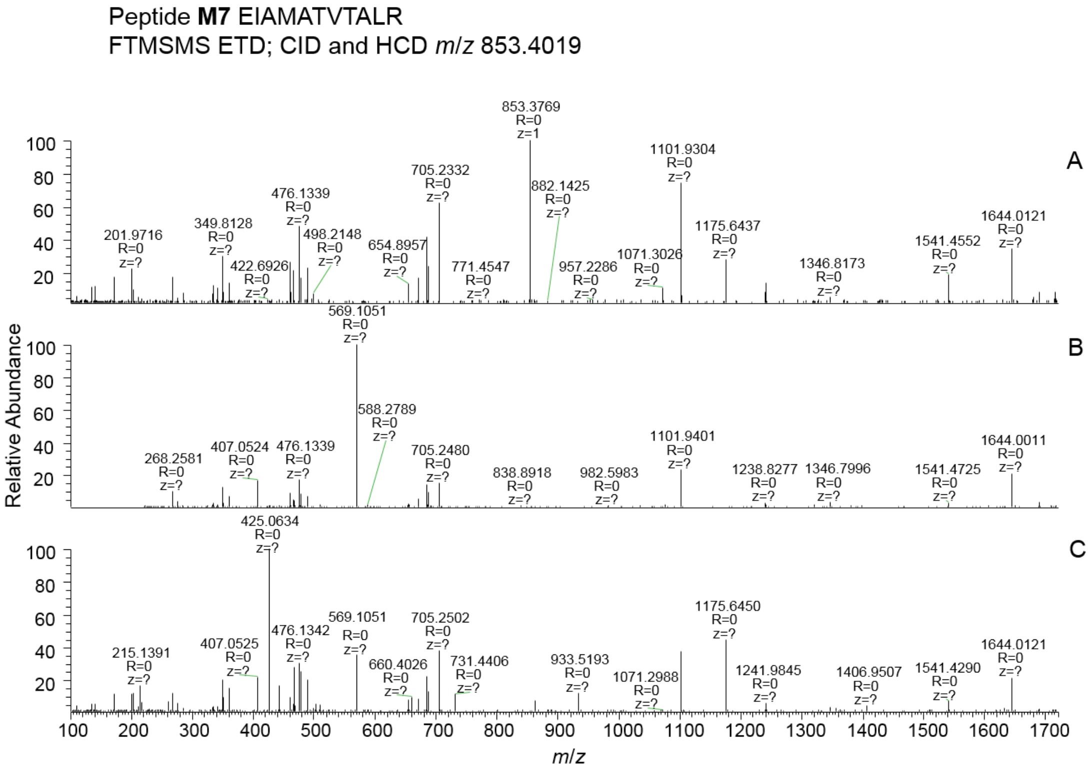

Figure S15: ESIMS/MS spectra of peptide M7. (A): ETD, (B): CID, (C): HCD.

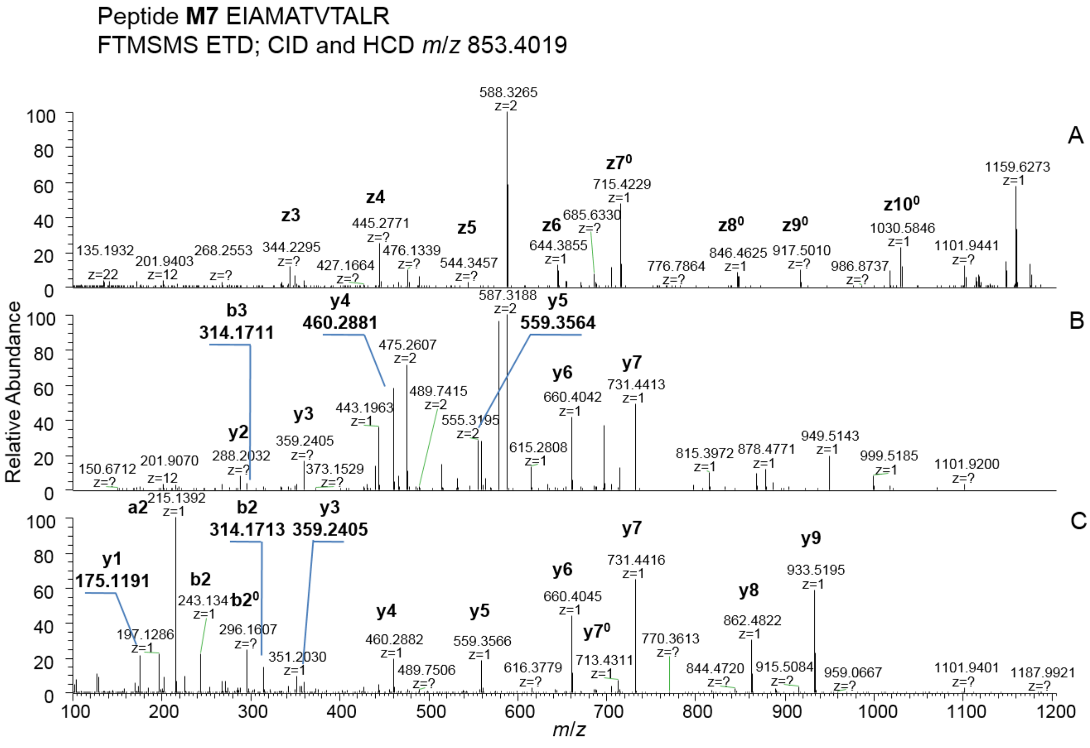

Figure S16: ESIMS/MS spectra of peptide M7 after labeling with **3**. (A): ETD, (B): CID, (C): HCD.

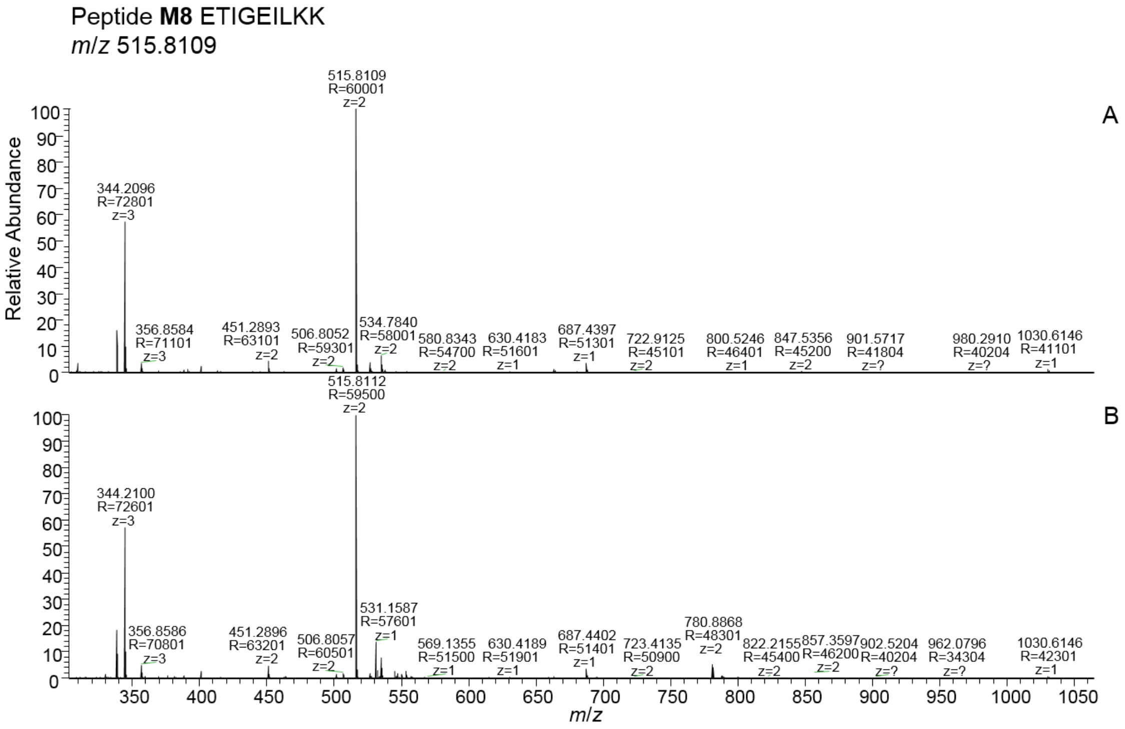

Figure S17: ESIMS spectra of peptide M8 before (A) and after (B) labeling with **3**.

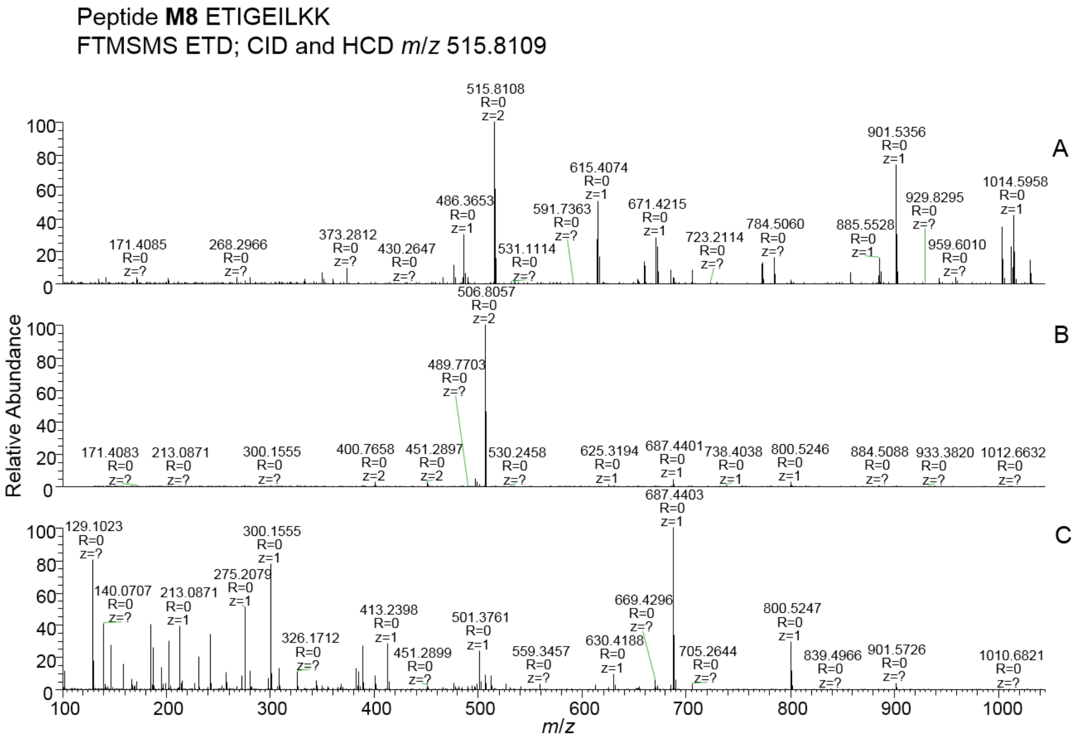

Figure S18: ESIMS/MS spectra of peptide M8. (A): ETD, (B): CID, (C): HCD.

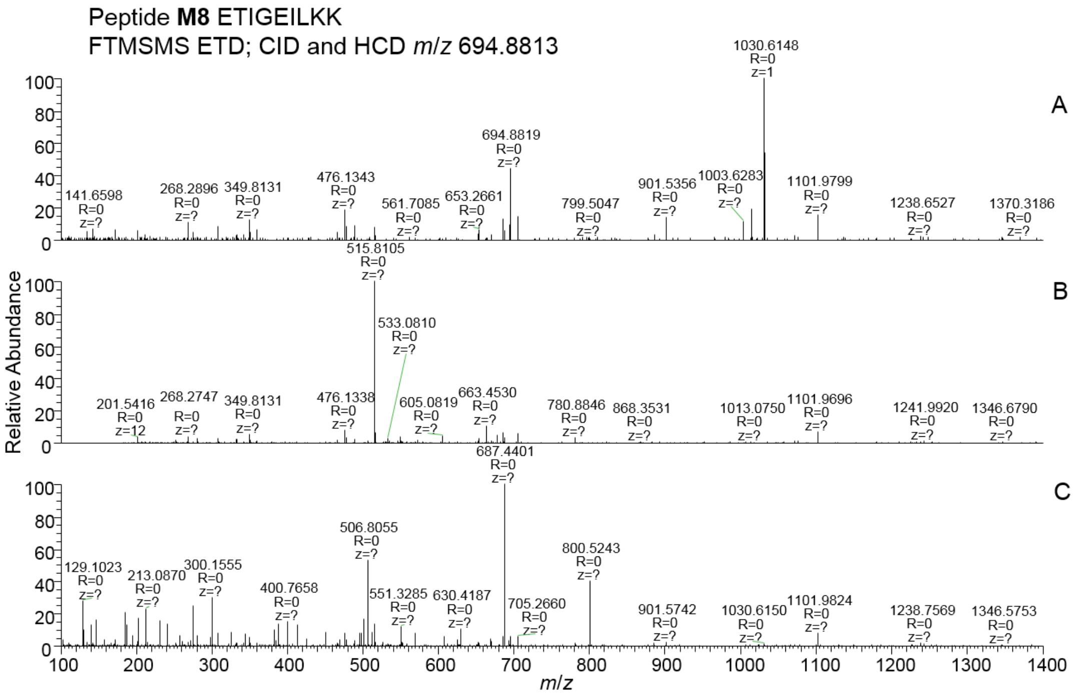

Figure S19: ESIMS/MS spectra of peptide M8 after labeling with **3**. (A): ETD, (B): CID, (C): HCD.

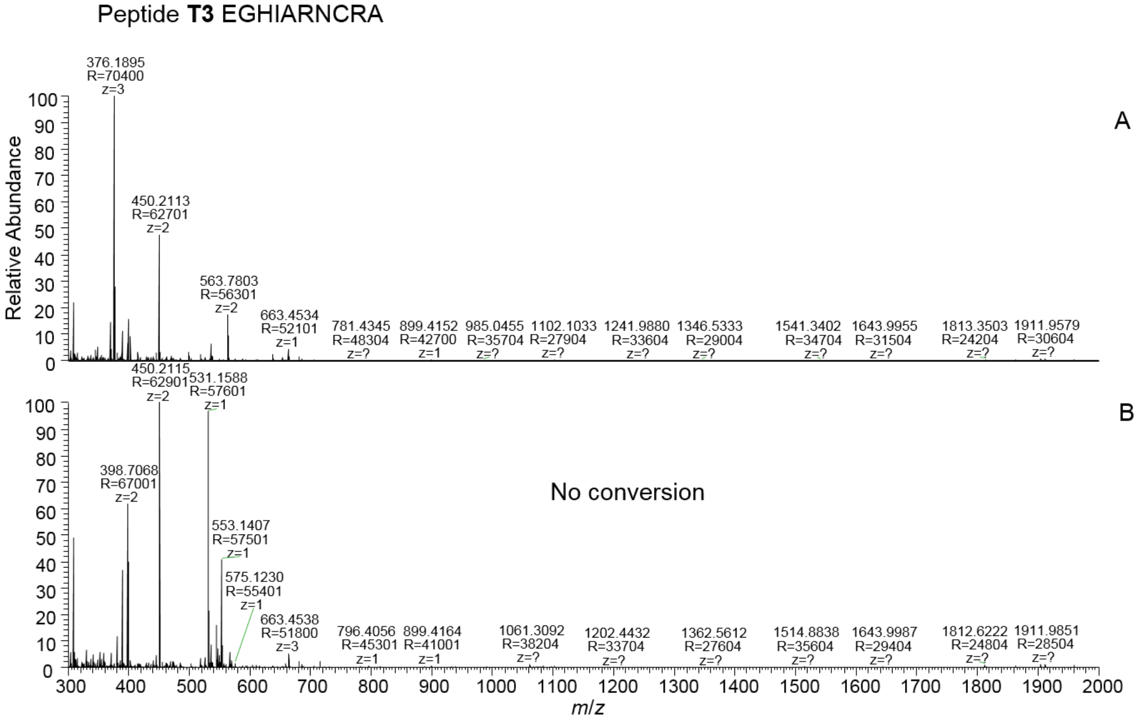

Figure S20: ESIMS spectra of peptide T3 before (A) and after (B) labeling with **3**.

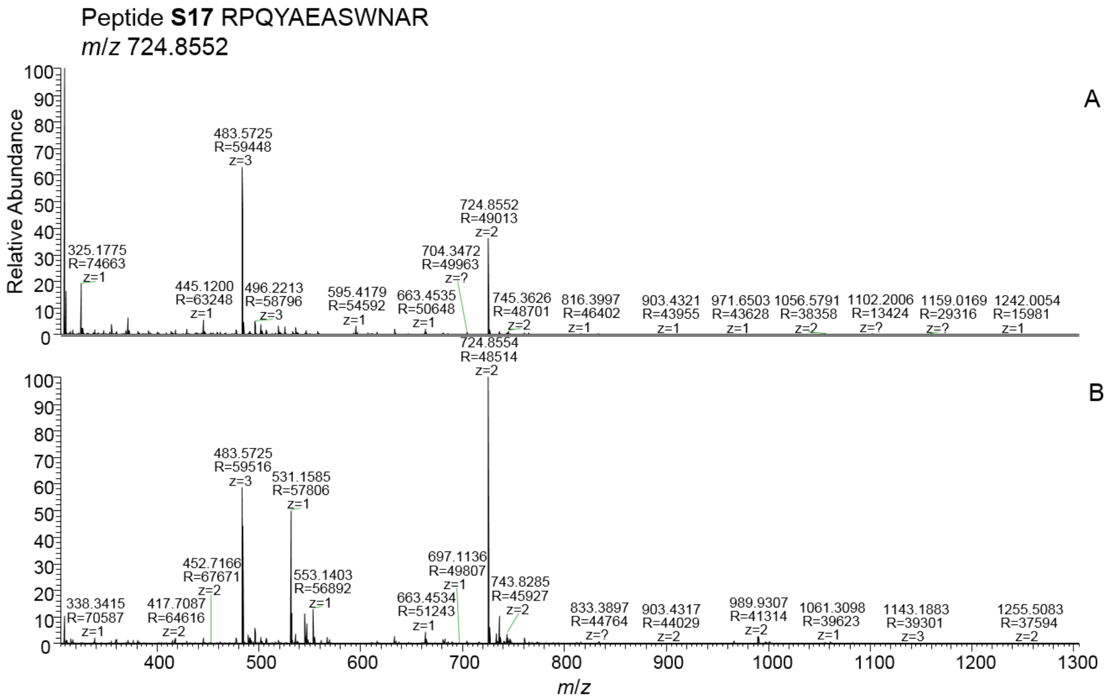

Figure S21: ESIMS spectra of peptide S17 before (A) and after (B) labeling with **3**.

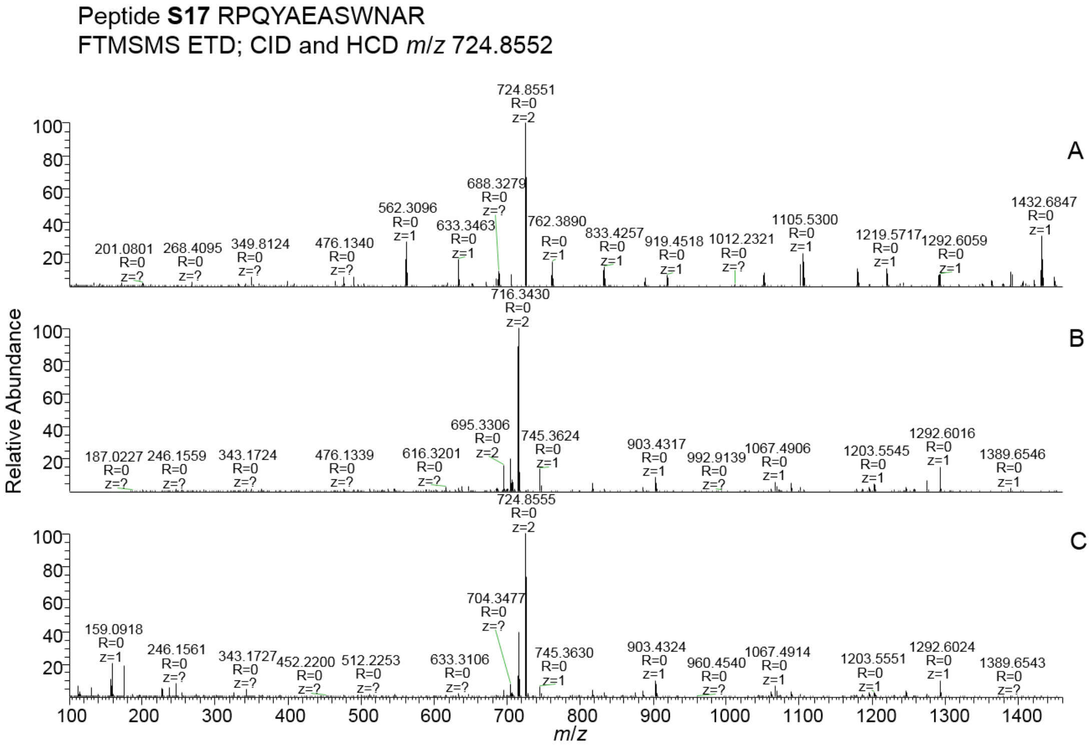

Figure S22: ESIMS/MS spectra of peptide S17. (A): ETD, (B): CID, (C): HCD.

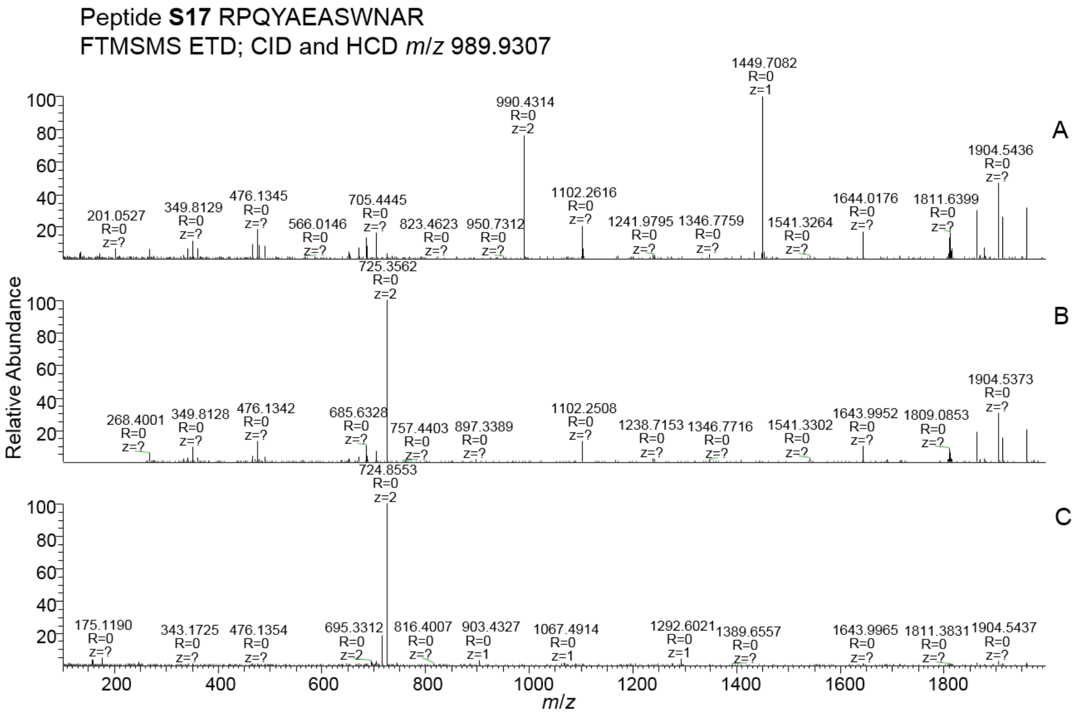

Figure S23: ESIMS/MS spectra of peptide S17 after labeling with **3**. (A): ETD, (B): CID, (C): HCD.

4. References

1. Kramer, B.; Metz, G.; Rarey, M.; Lengauer, T. *Med. Chem. Res.* **1999**, *9*, 463-478.

2. Rarey, M.; Kramer, B.; Lengauer, T.; Klebe, G. *J. Mol. Biol.* **1996**, *261*, 470-489.

3. Rarey, M.; Kramer, B.; Lengauer, T. *J. Comput. Aid. Mol. Des.* **1997**, *11*, 369-384.

4. Charifson, P. S.; Corkery, J. J.; Murcko, M. A.; Walters, W. P. *J. Med. Chem.* **1999**, *42*, 5100-5109.

5. Clark, R. D.; Strizhev, A.; Leonard, J. M.; Blake, J. F.; Matthew, J. B. *J. Mol. Graph. Model.* **2002**, *20*, 281-295.

6. Tripos, Inc., Sybyl 6.9, 1699 South Hanley Road, St. Louis, MO 63144-2319.

7. Hung, C. S.; Bouckaert, J.; Hung, D.; Pinkner, J.; Widberg, C.; Defusco, A.; Auguste, C. G.; Strouse, R.; Langermann, S.; Waksman, G.; Hultgren, S. J. *Mol. Microbiol.* **2002**, *44*, 903-915.

8. Bouckaert, J.; Berglund, J.; Schembri, M.; De Genst, E.; Cools, L.; Wuhrer, M., Hung, C. S., Pinkner, J.; Slättegard, R.; Zavialov, A.; Choudhury, D.; Langermann, S.; Hultgren, S. J.; Wyns, L.; Klemm, P.; Oscarson, S.; Knight, S. D.; De Greve, H. *Mol. Microbiol*. **2005**, *55*, 441-455.
